# Identification of Unanticipated and Novel *N*-Acyl L-Homoserine Lactones (AHLs) Using a Sensitive Non-Targeted LC-MS/MS Method

**DOI:** 10.1371/journal.pone.0163469

**Published:** 2016-10-05

**Authors:** Nishaben M. Patel, Joseph D. Moore, Helen E. Blackwell, Daniel Amador-Noguez

**Affiliations:** 1 Department of Bacteriology, 1550 Linden Dr., University of Wisconsin-Madison, Madison, Wisconsin, United States of America; 2 Master of Science in Bacteriology Program, University of Wisconsin-Madison, Madison, Wisconsin, United States of America; 3 Department of Chemistry, 1101 University Ave., University of Wisconsin-Madison, Madison, Wisconsin, United States of America; Laurentian, CANADA

## Abstract

*N*-acyl L-homoserine lactones (AHLs) constitute a predominant class of quorum-sensing signaling molecules used by Gram-negative bacteria. Here, we report a sensitive and non-targeted HPLC-MS/MS method based on parallel reaction monitoring (PRM) to identify and quantitate known, unanticipated, and novel AHLs in microbial samples. Using a hybrid quadrupole-high resolution mass analyzer, this method integrates MS scans and all-ion fragmentation MS/MS scans to allow simultaneous detection of AHL parent-ion masses and generation of full mass spectra at high resolution and high mass accuracy in a single chromatographic run. We applied this method to screen for AHL production in a variety of Gram-negative bacteria (i.e. *B*. *cepacia*, *E*. *tarda*, *E*. *carotovora*, *E*. *herbicola*, *P*. *stewartii*, *P*. *aeruginosa*, *P*. *aureofaciens*, and *R*. *sphaeroides*) and discovered that nearly all of them produce a larger set of AHLs than previously reported. Furthermore, we identified production of an uncommon AHL (i.e. 3-oxo-C7-HL) in *E*. *carotovora* and *P*. *stewartii*, whose production has only been previously observed within the genera *Serratia* and *Yersinia*. Finally, we used our method to quantitate AHL degradation in *B*. *cepacia*, *E*. *carotovora*, *E*. *herbicola*, *P*. *stewartii*, *P*. *aeruginosa*, *P*. *aureofaciens*, the non-AHL producer *E*. *coli*, and the Gram-positive bacterium *B*. *subtilis*. We found that AHL degradation ability varies widely across these microbes, of which *B*. *subtilis* and *E*. *carotovora* are the best degraders, and observed that there is a general trend for AHLs containing long acyl chains (≥10 carbons) to be degraded at faster rates than AHLs with short acyl chains (≤6 carbons).

## Introduction

Quorum sensing (QS) is a widespread form of cell-to-cell communication that allows bacteria to sense their surrounding population density and coordinately regulate a range of group-level behaviors, such as production of secondary metabolites and virulence factors, bioluminescence, and biofilm formation [1]. Bacterial QS involves production of signaling molecules known as autoinducers that are released into the extracellular environment [2]. In Gram-negative bacteria, the most prevalent QS system is the LuxI/LuxR QS system, which is based on the production of *N*-acyl L-homoserine lactone (AHL) signals [3–6]. In this system, LuxI protein homologs synthesize AHLs; once these signals reach a threshold concentration in a given environment (corresponding to a “quorate” population of cells), they productively bind to their cognate intracellular LuxR-type receptor/activator proteins to induce expression of QS regulated genes. AHLs are small diffusible signaling molecules characterized by a five-membered lactone ring and linear acyl tail of variable length (typically 4 to 14 carbons), which can have an oxo or hydroxyl group at the C-3 position (S1 Table). Over two dozen such AHL have been characterized to date, and there are likely more to be discovered [7–9].

The LuxI/LuxR QS system was initially discovered in the marine bacterium *Vibrio fischeri* [10,11]. Since then, AHL-dependent communication has been reported in many other gram-negative bacteria [12–18]. Screening for the presence of AHLs is commonly done with the aid of biosensor strains, which lack the LuxI homolog but contain an active LuxR-type receptor [19]. Upon binding of AHLs to the LuxR-type receptor, transcription of a reporter gene is induced, resulting in a phenotypic change (e.g., bioluminescence, β-galactosidase production, etc.). Recently, GFP-based biosensors in conjunction with thin-layer chromatography have resulted in improved sensitivity of AHL detection [20]. However, such biosensor strains can present challenges for novel AHL detection. Since they rely on the specificity of a given LuxR-type receptor for a particular AHL, biosensors are likely to miss detection of AHLs with different or novel structures. Additional limitations include their (i) failure to detect AHLs at concentrations below the activation threshold of the biosensors and (ii) general inability to provide accurate AHL quantitation. Therefore, more encompassing and sensitive methods beyond biosensor strains are needed to permit the detection and quantitation of the full set of AHLs produced by bacteria.

Liquid chromatography coupled to mass spectroscopy (HPLC-MS) can be a powerful technique to detect AHLs. For example, targeted LC-MS methods have been used to identify AHLs in *Nitrosomonas europaea*, *Pseudomonas aureofaciens*, *Pseudomonas fluorescens*, and *Pantoea stewartii* [15,21–24]. Furthermore, MS/MS analyses have proven useful in the characterization of AHLs with novel tail structures, such as an unsaturated aliphatic tail in *Methylobacterium extorquens* and *Rhodobacter sphaeroides* or the branched aliphatic tail in *Bradyrhizobium japonicum* [17,25,26]. However, despite the greater intrinsic sensitivity of MS, previous LC-MS methods utilized for AHL characterization have been targeted methods that rely on selected reaction monitoring (SRM) and therefore were not designed to screen for novel AHLs [24,27–29].

In the current study, we report a sensitive and non-targeted HPLC-MS/MS method based on parallel reaction monitoring (PRM) to identify and measure known, unanticipated, and novel AHLs in microbial samples. Making use of a hybrid quadrupole-orbitrap mass analyzer, the analytical method presented here integrates full-scan MS with successive all-ion fragmentation (AIF) MS/MS scans to allow parallel detection of AHL parent-ion masses and full mass spectra at high resolution and high mass accuracy within a single chromatographic run. We applied this method to screen for AHL production in a variety of Gram-negative bacteria under different growth conditions. In nearly all of the bacteria examined, we observed unanticipated production (i.e., not previously reported in the literature) of known AHLs. We also utilized this new MS-based method to investigate and quantitate AHL degradation capabilities across diverse bacteria.

## Results

### Development of a non-targeted LC-MS/MS method for detection of unanticipated and novel AHLs

#### Quantitation of native and non-native AHLs by LC-MS

As the initial component of a non-targeted method for the identification of unknown AHLs, we first developed a sensitive non-targeted LC-MS method for detection and quantitation of native and non-native AHLs standards. We used ultra-high-pressure reverse-phase liquid chromatography coupled to a high-resolution mass spectrometer operating in full-scan mode. HPLC was coupled to MS via positive electrospray ionization (+ESI). Under these conditions, the dominant pseudo-molecular ion observed for all AHL standards was [M + H]^+^. Identification of each AHL was established based on its retention time and the exact mass of its singly protonated species.

We used a combination of 23 native and non-native AHL standards to determine limits-of-detection (LOD) and chromatographic retention times (S1 Table). Non-native AHLs contained structurally diverse variations to the basic AHL structure, such as aryl or branched aliphatic tails, and were included to broaden the scope of our method to AHLs with unconventional structures. In addition, we also included in our analyses a compound (mBTL) with close structural similarity to AHLs but containing a thiolactone ring instead of a lactone ring (S1 Table) [30].

**Fig 1:**
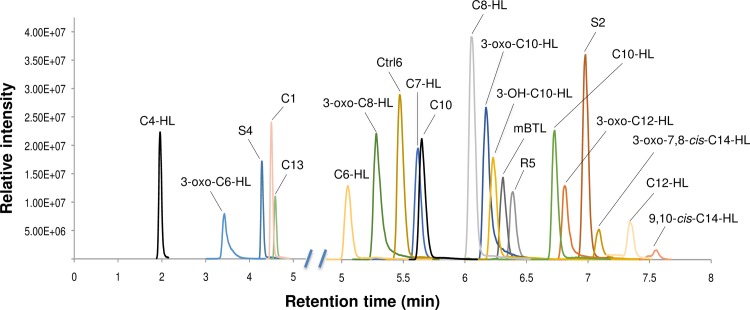
Extracted Ion chromatographs of AHL standards. The ion counts of each pseudo-molecular ion [M + H]^+^ are shown against retention time. Retention time axis is broken to expand the scale after five minutes, where most AHLs elute. Range for concentration of AHLs was between 0.04 μM to 3.70 μM. Compound structures are shown in S1 Table.

Our chromatographic method yielded good separation and sharp/defined peaks for nearly all of the AHL standards (Fig 1). In Table 1, we summarize the results of the LC-MS method validation. The mass accuracy for all standards was within 3 ppm (parts per million). The median LOD was 2.3 nM, with 19 AHL standards having a LOD of less than 15 nM. The LOD was established as the lowest concentration of the standard at which signal was at least 3 times higher than background noise. Linearity was examined over a 100-fold concentration range; the median R^2^ value was >0.99, indicating a good linear response (S1 Fig). The quantitative reproducibility (intra-day), tested at a median compound concentration of 0.1 uM, had a median value of ~7% (Table 1).

**Table 1:** Limits of detection and retention times of AHL standards.*

		Limit of detection						
Standard*	Property	nM**	pg	Theoretical mass ***	Observed mass	Retention time (min)	R^2^	RSD
C4-HL	Native	11.3	7.81	172.097	172.097	1.9	0.996	3.043
C6-HL	Native	2.0	1.56	200.128	200.128	5.0	0.999	7.900
3-oxo-C6-HL	Native	0.9	0.78	214.107	214.107	3.4	0.997	8.416
C7-HL	Native	0.6	0.55	214.144	214.144	5.6	0.999	6.944
C8-HL	Native	8.6	7.81	228.159	228.159	6.0	0.999	6.881
3-oxo-C8-HL	Native	8.1	7.81	242.139	242.138	5.3	1.000	8.830
C10-HL	Native	0.6	0.64	256.191	256.190	6.7	0.999	7.666
3-OH-C10-HL	Native	229.6	250	272.186	272.185	6.2	1.000	5.025
3-oxo-C10-HL	Native	14.5	15.63	270.170	270.170	6.1	1.000	5.506
C12-HL	Native	219.9	250	284.222	284.222	7.3	0.959	5.966
3-oxo-C12-HL	Native	2.6	3.13	298.201	298.201	6.8	0.987	8.899
9,10-*cis*-C14-HL	Native	805.8	1000	310.238	310.237	7.5	0.911	6.062
3-oxo-7,8-*cis*-C14-HL	Native	385.5	500	324.217	324.217	7.0	0.981	6.167
3-oxo-11,12-*cis*-C16-HL	Native	2838.9	4000	352.248	352.248	7.5	0.947	3.818
9,10-*cis*-C18-HL	Native	10.7	15.63	366.300	366.300	9.6	0.976	9.918
C1	Non-native	0.9	0.78	220.097	220.097	4.5	0.999	6.186
C2	Non-native	0.8	0.78	238.087	238.087	4.7	0.997	7.199
C10	Non-native	0.6	0.78	345.994	345.993	5.6	0.998	5.347
C13	Non-native	0.7	0.78	265.082	265.082	4.5	1.000	7.313
S2	Non-native	2.7	3.13	284.222	284.222	6.9	0.995	8.293
S4	Non-native	1.0	0.78	198.112	198.112	4.3	1.000	7.120
R5	Non-native	0.7	0.78	292.154	292.154	6.4	0.999	7.214
Ctrl 6	Non-native	0.8	0.78	248.128	248.128	5.6	1.000	7.127
mBTL	Non-AHL	0.5	0.78	358.011	358.010	6.3	1.000	7.863

* See S1 Table for molecular structures.

** Using a 4 μL injection volume.

*** Theoretical mass corresponds to the pseudo-molecular ion [M + H]+.

R^2^: correlation coefficient for linearity of AHL measurements over a 100-fold concentration range (see also S1 Fig).

RDS: Relative standard deviation (intra-day), tested at a median compound concentration of 0.1 μM.

#### AHL fragmentation spectra library

**Fig 2:**
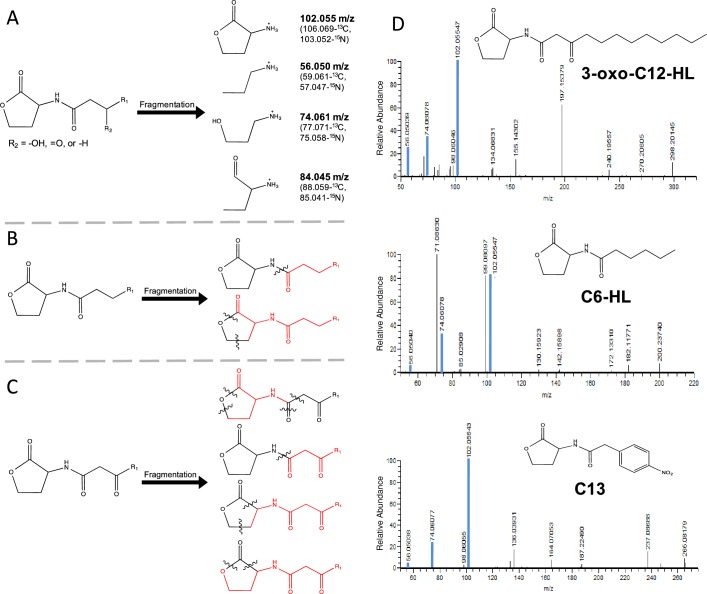
Characteristic MS/MS fragmentation of AHLs. A) Characteristic fragments originating from the lactone ring. These fragments were used to identify AHLs in bacterial samples. Labeled masses for carbon (^13^C) and nitrogen (^15^N) isotopes are indicated in parenthesis. B) Characteristic fragmentation observed when no substitution is present at the third carbon of the acyl chain. C) Characteristic fragmentation observed when a 3-oxo substitution is present. In B) and C), black wavy lines indicate the bonds that are broken during fragmentation, the measured fragment is highlighted in red. D) Fragmentation spectra of selected AHL standards. Blue lines indicate peaks for characteristic fragments of the lactone ring.

We next generated a MS/MS fragmentation spectra library to serve as the basis for non-targeted detection of AHLs. Based on analyses of fragmentation spectra of native and non-native AHLs standards, we identified four characteristic fragments originating from the lactone ring in AHLs (Fig 2A). The masses for these species were 102.055, 84.045, 74.061, and 56.050 m/z. The 102.055 m/z fragment corresponds to the lactone ring itself, while the rest were characteristic fragments arising from fragmentation of the lactone ring. In addition to these fragments, we identified other common fragmentation patterns in AHL standards. As shown in Fig 2B and 2C, these fragmentation patterns differed depending on whether or not an oxo group was present at the third carbon in the acyl chain of AHLs. Fragmentation spectra of selected AHL standards are shown in Fig 2D (the full set of spectra is shown in S2 Fig).

**Fig 3:**
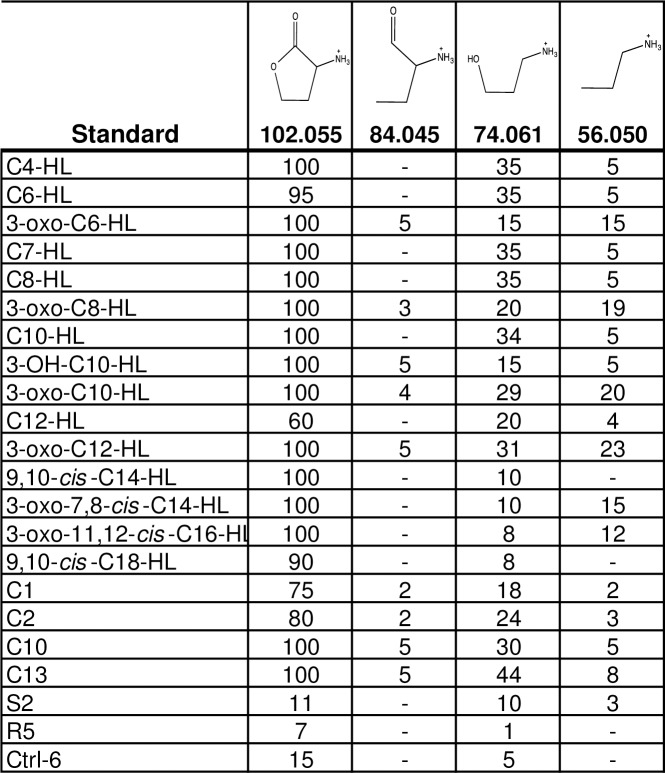
Relative abundance of characteristic lactone ring fragments obtained from MS/MS spectra of AHL strandards.

As shown in Fig 3, all of the native AHL standards, and nearly all of the non-native AHL standards, displayed a prominent 102.055 m/z fragment. The second and third most prominent fragments were lactone fragments, present also in nearly all AHL standards, with 74.061 m/z and 56.050 m/z, respectively. The compound mBTL, a non-AHL standard with a thiolactone ring, produced a 118 m/z fragment, corresponding to the thiolactone ring and analogous to the 102.055 m/z species originating from the lactone ring in AHLs.

#### LC-MS/MS detection of unanticipated and novel AHLs

Our next goal was to develop a non-targeted method for AHL detection based on the characteristic fragmentation patterns of the lactone ring. We designed our method to perform an MS1 full-scan (100 to 510 m/z, no fragmentation) together with a series of MS/MS scans (all-ion fragmentation) that divided the m/z range into partially overlapping windows of 40 m/z each. The MS1 full-scan provides data on [M + H]^+^ pseudo-molecular ions, while the MS/MS scans provide corresponding (matched by retention time) fragmentation spectra, all obtained within a single chromatographic run.

Using this method, identification of known AHLs is based on their retention time, the exact mass of their [M + H]^+^ pseudo-molecular ions, and their characteristic MS/MS fragmentation pattern. In turn, the identification of unanticipated/novel AHLs is performed by identifying characteristic fragments of the lactone ring in MS/MS scans. The lactone ring fragment (102.055 m/z) and at least one other characteristic lactone ring fragment have to be present and co-elute for a molecule to be considered as a potentially novel AHL. Candidates of the parent ion of the putative AHL can then be identified from the MS full-scan. Final confirmation is then performed by an additional chromatographic run where potential parent ions are isolated and analyzed via parallel reaction monitoring (PRM).

AHL quantitation can be performed using either the signal (extracted ion chromatogram peak area) of the [M + H]^+^ pseudo-molecular ion obtained from the MS1 scan or using the signal from the lactone ring fragment (102.055 m/z) obtained from the MS2 scan. While nearly all standards could be quantitated based on the exact masses of their pseudo-molecular ion without any significant interference, we did observe a low-level contaminant with the same mass and retention time as 3-OH-C10-HL that interfered with accurate quantitation. Our HPLC-MS/MS method allowed for identification of 3-OH-C10-HL and its differentiation from the contaminant based on its AHL characteristic fragments. Therefore, 3-OH-C10-HL could be quantified based on its lactone ring fragment (102.055 m/z) rather than its [M + H]^+^ pseudo-molecular ion.

### Detection of known and unanticipated AHLs in microbial samples

#### Quantitation of AHLs in bacterial samples

To test our non-targeted LC-MS/MS method, we followed AHL production under different growth conditions (e.g., different carbon sources) in a variety of Gram-negative bacteria: *Burkholderia cepacia*, *Edwardsiella tarda*, *Erwinia carotovora*, *Erwinia herbicola*, *Pantoea stewartii*, *Pseudomonas aeruginosa*, *Pseudomonas aureofaciens*, and *Rhodobacter sphaeroides*. All bacteria tested were known AHL producers [15,17,22,31–36]. AHL quantitation was performed by using two non-native AHLs, S2 and S4 (S1 Table), as internal standards that were added to spent media samples before performing liquid to liquid extraction (see Materials and Methods section).

Table 2 displays a summary of our results. In addition to detecting previously reported AHLs [4,13,15,17,22,31–39], we detected many unanticipated AHLs in all of the bacteria tested. For example, while *B*. *cepacia* is known to only produce C6-HL and C8-HL [10], we observed eight additional AHLs: C4-HL, C7-HL, 3-oxo-C8-HL, 3-OH-C8-HL, C10-HL, 3-oxo-C10-HL, 3-OH-C10-HL, and 3-oxo-C12-HL. Similarly, *E*. *carotovora* and *P*. *stewartii* are known to only produce 3-oxo-C6-HL [4], but we observed the production of six and seven additional AHLs in these bacteria, respectively. These findings indicate that many AHLs are yet to be recognized even in microbes that have been previously screened for AHLs. Further, these results highlight the sensitivity of our non-targeted LC-MS/MS method.

**Table 2:** AHL detection in bacterial samples.*

Bacterium	Expected AHL	Observed AHL (unanticipated in bold)^†^	
*Burkholderia cepacia AMMO*	C8-HL	C8-HL	**C10-HL**
	C6-HL	C6-HL	**3-oxo-C8-HL**
		**3-oxo-C10-HL**	**3-oxo-C12-HL**
		**C7-HL**	**C4-HL**
		**3-OH-C8-HL**	**3-OH-C10-HL**
*Edwardsiella tarda*	C4-HL	C4-HL	**C8-HL**
	C6-HL	C6-HL	**3-oxo-C8-HL**
	C7-HL	3-Oxo-C6-HL	
	3-oxo-C6-HL		
*Erwinia carotovora*	3-oxo-C6-HL	3-oxo-C6-HL	**3-oxo-C6-HL**
		**3-oxo-C10-HL**	**3-oxo-C10-HL**
		**3-oxo-C8-HL**	**C7-HL**
			**3-oxo-C7-HL**
*Erwinia herbicola LS005*	C4-HL	C4-HL	**3-oxo-C10-HL**
		**C8-HL**	
*Pantoea stewartii*	3-oxo-C6-HL	3-oxo-C6-HL	**C7-HL**
		**3-oxo-C8-HL**	**C8-HL**
		**C6-HL**	**3-oxo-C10-HL**
		**C4-HL**	**3-oxo-C7-HL**
*Pseudomonas aeruginosa PAO1*	3-oxo-C12-HL	3-oxo-C12-HL	3-OH-C10-HL
	C4-HL	C4-HL	3-oxo-C6-HL
	C6-HL	C6-HL	**3-oxo-C10-HL**
	C8-HL	C8-HL	**3-oxo-C8-HL**
	3-OH-C10-HL		
	3-OH-C12-HL		
	3-oxo-C6-HL		
*Pseudomonas aureofaciens 30–84*	C6-HL	C6-HL	**3-OH-C8-HL**
		**C4-HL**	**3-oxo-C10-HL**
*Rhodobacter sphaeroides 2*.*4*.*1*	7,8-*cis*-C14-HL	7,8-*cis*-C14-HL	**C14-HL**
		**3-oxo-C14-HL**	**3-OH-C14-HL**

*AHL concentrations are shown in S2 Table.

^†^Previously unreported AHLs in each bacterium are shown in bold.

We found that AHL production levels varied considerably within and across microbes (S2 Table). Measured AHL concentrations were as high as ~50 μM (for C4-HL in *P*. *aeruginosa*) to as low as ~1 nM (for C7-HL in *P*. *stewartii*). While most of the unanticipated AHLs were produced at low concentrations, explaining at least in part why they may not have been detected previously, a few of them were produced at higher levels than previously reported AHLs. For example, *E*. *carotovora* produced three unanticipated AHLs: 3-oxo-C10-HL, 3-oxo-C8-HL, and C8-HL, in higher amounts (~1, 4, and 10 μM, respectively) than the previously reported 3-oxo-C6-HL signal (~0.2 μM) [4]. Similarly, in *P*. *aureofaciens*, C4-HL, an unanticipated AHL for this bacterium, was produced at higher levels (~5 μM) than C6-HL (~10 nM), the previously reported native AHL signal [22].

#### Detection of 3-oxo-C7-HL in *P*. *stewartii* and *E*. *carotovora*

Our method was developed for non-targeted analysis of AHLs, and it is therefore capable of identifying unknown AHLs. We sought to test this capability. Accordingly, we screened for the presence of novel AHLs in spent media samples (from all of the bacteria listed in Table 2) by identifying co-eluting characteristic fragments of the lactone ring (e.g., 102.055, 84.045, 74.061, and 56.050 m/z) in MS/MS scans. Our first criteria for the identification of putative novel AHLs was the presence of a prominent 102.055 m/z peak in MS/MS scans and at least one other characteristic lactone ring fragment that could not be matched to the pseudo-molecular ion mass (given by MS1 scans) and retention time of any known AHLs.

Although we did not identify AHLs with novel structures in any of the bacteria that we screened, we detected production of a rare AHL, 3-oxo-C7-HL, in *E*. *carotovora* and *P*. *stewartii*. Production of 3-oxo-C7-HL has only been previously reported in *Serratia plymuthica* and *Yersinia pseudotuberculosis* [40–42]. We used the detection and MS/MS characterization of this uncommon AHL as an example of how our method would work at identifying novel AHLs. MS1 scans identified 228.123 m/z as the putative parent ion corresponding to 3-oxo-C7-HL. To show that this [M + H]^+^ pseudo-molecular ion corresponded to 3-oxo-C7-HL, we performed an additional targeted MS/MS fragmentation analysis on this ion that corroborated the appearance of the characteristic lactone ring fragments 102.055 and 74.061 m/z. The exact mass of the [M + H]^+^ pseudo-molecular ion, 228.123 m/z, can be matched to a single molecular formula: C_11_H_18_NO_4_, which corresponds to 3-oxo-C7-HL, an AHL with a tail of seven carbons and an oxo group at carbon-3 position.

**Fig 4:**
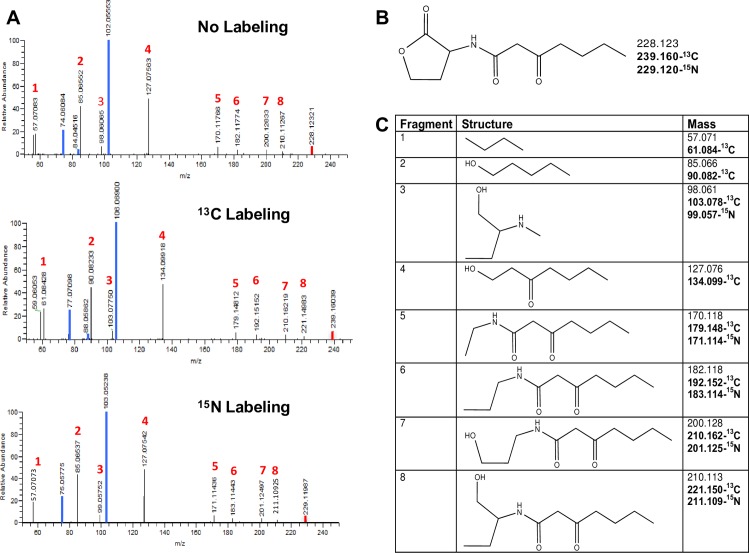
3-oxo-C7-HL detection in *P*. *stewartii*. (A) Fragmentation spectra when bacteria are grown in medium with no labeled components, medium with 100% uniformly labeled glucose (^13^C labeling), and medium with labeled ammonium (^15^N labeling). Characteristic lactone ring fragments are highlighted in blue and parent ion is highlighted in red. (B) Structure of 3-oxo-C7-HL and its ^12^C, ^13^C, and ^15^N masses. (C) Structures and masses of fragments of 3-oxo-C7-HL in non-labeled, ^13^C-labeled, and ^15^N-labeled media. Similar results were observed for *E*. *carotovora*.

Confirmation of the molecular formula and structure of 3-oxo-C7-HL was based on MS/MS fragmentation spectra in combination with ^13^C-carbon and ^15^N-nitrogen labeling (Fig 4). When *P*. *stewartii* and *E*. *carotovora* were grown on uniformly labeled ^13^C-glucose as its sole carbon source, the mass of the putative AHL (3-oxo-C7-HL) shifted from 228.123 to 239.160. This represented a difference of 11.037 mass units that confirmed the presence of 11 carbons in this molecule. Similarly, when cells were grown on ^15^N-ammonia, we observed a mass shift from 228.123 to 229.120, a difference of 0.997 mass units that confirmed the presence of a single nitrogen. We used the MS/MS spectra obtained in non-labeled, ^13^C-labeled, and ^15^N-labeled media to corroborate the structure of this AHL. The MS/MS spectra in non-labeled media displayed a dominant 102.055 peak corresponding to the lactone ring and another prominent characteristic lactone ring fragmentation peak: 74.061. Based on their exact mass and the mass shifts observed in ^13^C- and ^15^N-media, we deduced molecular formulas and structures for the rest of the fragments in the mass spectrum. In agreement with our AHL fragmentation library, the rest of the peaks in the spectrum corresponded to the expected AHL fragments originating from the tail alone or from the tail plus a fragment of the lactone ring. The identity of all MS/MS peaks was further confirmed by the spectra obtained from ^13^C-labeled and ^15^N-labeled samples, which respectively corroborated the number of carbons and the presence of nitrogen in each fragment (Fig 4C).

Since a standard for 3-oxo-C7-HL was not available, we estimated its concentration based on the average response factors of 3-oxo-C6-HL and 3-oxo-C8-HL. Based on this approximation, 3-oxo-C7-HL production was ~240 nM and ~10 nM in *E*. *carotovora* and *P*. *stewartii*, respectively.

Comparison of spectra of 3-oxo-C7-HL against AHLs with similar structures (3-oxo-C6-HL, 3-oxo-C8-HL, and C7-HL) revealed that, as would be expected, 3-oxo-C7-HL had a fragmentation pattern more similar to 3-oxo-C6-HL and 3-oxo-C8-HL than to C7-HL (S4 Fig). For example, 102.055 m/z corresponded to the largest peak in the spectra of 3-oxo-C6-HL, 3-oxo-C7-HL, and 3-oxo-C8-HL, while a non-lactone ring fragment was the predominant peak in C7-HL.

### AHL degradation

#### Detection of hydrolyzed AHLs

Degradation of AHLs by AHL-lactonase enzymes, which hydrolyze the lactone bond to produce corresponding *N*-acyl homoserines, is an important and debated mechanism of “quorum quenching” in bacteria [3,19,43–53]. We considered that our non-targeted LC-MS/MS method could also be used for monitoring this type of enzymatic degradation. To evaluate this, we tested our method on our set of AHL standards after they were hydrolyzed by incubation in 1 M NaOH.

We found that hydrolysis of the lactone ring can be readily assessed by our method without any modifications (S5 Fig). AHL hydrolysis could be determined by: i) an increase in the mass of the [M + H]^+^ pseudo-molecular ion by 18.011 mass units (corresponding to water addition), ii) the appearance of a characteristic 120.065 m/z peak in the MS/MS spectrum that corresponded to the hydrolyzed lactone ring fragment, and ii) a small shift (~10 s) towards a shorter retention time. Interestingly, we found that all hydrolyzed AHLs still displayed a prominent 102.055 peak and other characteristic lactone ring fragment peaks in their mass spectra, which we attribute to loss of water and reconstitution of the lactone ring during fragmentation in the mass spectrometer.

We observed that AHL hydrolysis occurs naturally in some of the bacterial cultures that we analyzed (i.e., *B*. *cepacia* and *R*. *spharoides*). Specifically, *B*. *cepacia* cultures displayed significant hydrolysis of C6-HL during stationary phase (up to ~40% hydrolysis). Similarly, significant hydrolysis of 7,8-*cis*-C14-HL (~70% hydrolysis) was observed in stationary phase *R*. *spharoides* cultures.

#### AHL degradation by bacteria

Finally, we used our LC-MS/MS method to investigate AHL degradation by different AHL producers: *B*. *cepacia*, *E*. *carotovora*, *E*. *herbicola*, *P*. *stewartii*, *P*. *aureofaciens*, and *P*. *aeruginosa;* and in two non-AHL producing bacteria: the Gram-positive bacterium *B*. *subtilis* and the Gram-negative bacterium *E*. *coli*. Many of these bacteria are reported to contain AHL degrading enzymes, such as AHL lactonases or AHL acylases, that cleave the lactone ring or the AHL amide, respectively [47,54]. To test the AHL degradation capabilities of these bacteria, we added a mix of 15 AHL standards (both native and non-native at 1 μM each) to mid-exponential cultures and monitored AHL degradation over time. To discriminate between degradation of endogenously produced AHLs vs. degradation of added AHL standards, all AHL producers were grown in media with ^15^N-labeled ammonia, which labeled all endogenously produced AHLs. Media without cells was used as a control to correct for spontaneous degradation.

**Fig 5:**
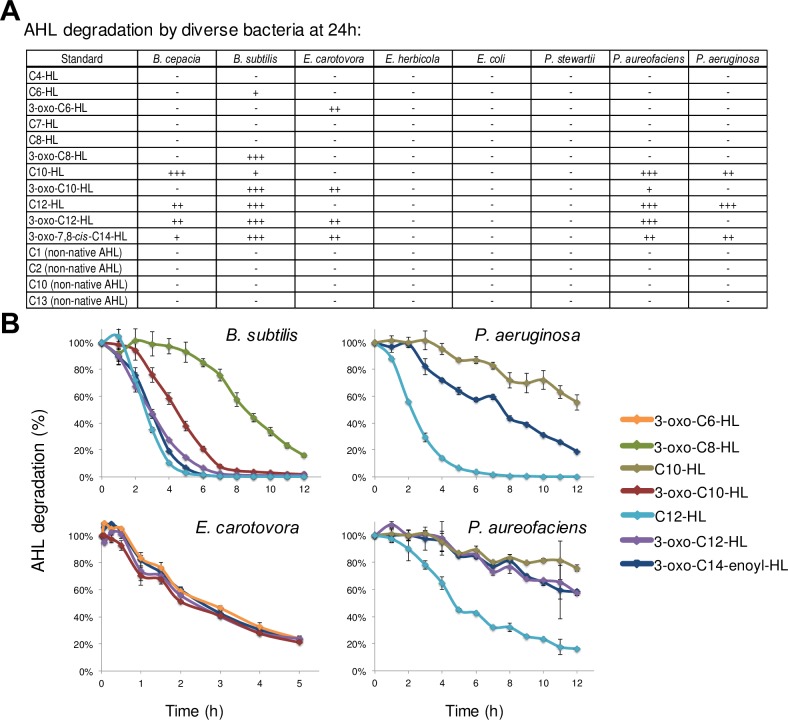
AHL degradation by bacteria. (A) AHL standards were added at 1 μM concentration to exponentially growing cultures and AHL degradation was measured after 24 hours. The amount of AHL degradation at 24 hours is shown as: +++, >90% degradation; ++ 60–90% degradation; +, 30–60% degradation; -, less than 30% degradation. (B) Dynamic AHL degradation in selected bacteria. AHLs (10 μM each) were added to exponentially growing cultures and AHL degradation was monitored over time. Data represents the average of three biological replicates.

We observed wide variability in the AHL degradation abilities of the tested bacteria (Fig 5A). By 24 hours, we observed significant AHL degradation by *B*. *cepacia*, *B*. *subtilis*, *E*. *carotovora*, *P*. *aureofaciens*, and *P*. *aeruginosa*. Interestingly, *E*. *herbicola*, *P*. *stewartii*, and *E*. *coli* did not appreciably degrade any AHLs. Among the bacteria that displayed AHL degradation capabilities, we observed a general preference towards the degradation of long tail AHLs vs. short tail AHLs (Fig 5A). For example, only *E*. *carotovora* was capable of degrading the short tail 3-oxo-C6-HL; and none of the tested bacteria were capable of degrading C4-HL, C6-HL, C7-HL, or C8-HL. In contrast, several bacteria were capable of degrading longer tail AHLs such as C10-HL, 3-oxo-C10-HL,3-oxo-C12-HL, C12-HL, and others. Interestingly, none of the microbes were capable of degrading any of the non-native AHLs tested (Fig 5A). We did not find a measurable accumulation of hydrolyzed AHLs in any of these experiments, indicating that AHL degradation proceeds beyond simple hydrolysis of the lactone ring in all degrading bacteria.

Fig 5B shows a detailed AHL degradation profile for some of the best degraders (i.e., *B*. *subtilis*, *E*. *carotovora*, *P*. *aureofaciens*, and *P*. *aeruginosa*). AHLs against which these microbes displayed the best degradation were added at 10 μM concentrations (non-native AHL C2 was added as a negative control). All of these microbes, with the exception of *E*. *carotovora*, displayed different degradation rates for different AHLs. For example, *B*. *subtilis* degraded 3-oxo-7,8-cis-C14-HL, C12-HL, and 3-oxo-C12-HL, at higher rates than 3-oxo-C8-HL; *P*. *aeruginosa* degraded C12-HL faster than 3-oxo-7,8-cis-C14-HL or C10-HL. In contrast *E*. *carotovora* degraded AHLs at nearly the same rate.

We were interested in correlating the observed AHL degradation capabilities of AHL degrading bacteria with the presence of known AHL degrading enzymes (i.e., AHL-lactonases and AHL-acylases) [47]. *P*. *aeruginosa* is known to contain at least two AHL-acylases: PvdQ and QuiP [55,56]. PvdQ and QuiP are both reported to preferentially degrade AHLs with long acyl-chains, which agrees well with our results [54,55]. Additionally, *P*. *aeruginosa* contains genes for two other putative acylases, *pa1893* and *pa0305*. While *pa0305* encodes an effective acylase that has activity against long-chain AHLs, not much is known regarding the function of *pa1893* [57]. *B*. *subtilis* is known to possess a AHL-lactonase, AiiA, which was shown to have activity against various AHLs [49,58,59]. AHL degrading enzymes have not been identified in *B*. *cepacia*, *E*. *carotovora*, or *P*. *aureofaciens*. A genome search revealed that *B*. *cepacia* possesses a homologue gene (Genbank accession CP003775) with 74% similarity to AttM, an AHL-lactonase from *Agrobacterium tumefaciens* [60]. In *P*. *aureofaciens*, we found a homologue gene (Genbank accession WP_041984058) with 58% similarity to *P*. *aeruginosa*’s PvdQ [55]. We did not find any lactonase or acylase homologues in *E*. *carotovora*, despite the AHL degradation activity displayed by this microbe.

## Discussion

We report herein a non-targeted HPLC-MS/MS method for detecting and quantitating AHLs. This method identifies AHLs based on the characteristic MS/MS fragmentation pattern of the lactone ring and can therefore be employed towards screening bacterial samples for novel AHLs. As we have shown, our method can also be used to investigate AHL degradation by bacteria. The limits of detection for most AHLs are in the low nanomolar range, which is lower than typical levels of AHLs found in bacterial samples (0.1–10 μM) [22]. This threshold provides confidence that our method will detect, in a non-biased manner, most of the AHLs produced by any given bacteria, provided that the right growth conditions for AHL production are fulfilled. Although we used liquid-liquid extraction prior to AHL measurement in most of the experiments shown here, our method is sensitive enough that quantitation of predominant AHLs can be performed directly from a small volume (10 μL) of spent media without the need for extraction or sample concentration.

We note that other methods have been reported recently that utilize LC-MS/MS for quantitation of AHLs; however, they have been SRM-based methods that do not easily allow for the non-targeted identification of AHLs [24,27–29]. The method reported here also delivers a higher confidence in AHL identification relative to these prior methods by providing high-resolution AHL mass spectra. Even though our method was developed using a hybrid quadrupole/orbitrap-mass-analyzer, it can be readily implemented on other commonly available platforms such as hybrid quadrupole/time-of-flight mass spectrometers. As such, we believe that it could be adopted in a range of research settings.

### Detection of AHLs in bacterial samples

We applied our non-targeted LC-MS/MS method to quantitate AHL production in a variety of Gram-negative bacteria under different growth conditions. In addition to detecting AHLs previously known to be produced by each of these bacteria, we also identified unanticipated AHLs in most of them (Table 2). One possible reason for detecting numerous unanticipated AHLs may be the high sensitivity of our method, which allows for the detection of AHLs even when present at low concentrations. Also, a non-targeted screening such as the one used in this study has not been performed before in many of the tested bacteria, and therefore such diversity of AHL production was not previously observed [4,13,15,17,22,31–39]. Based on these findings, we consider likely that many Gram-negative bacteria produce a larger number of AHLs than reported so far in the literature. Our results also showed that for any given microbe, the same set of AHLs was produced across all growth conditions tested; variations in AHL levels were correlated to final cell density across all media conditions. The ability of most of the unanticipated AHLs that we report here at activating biosensors has been previously demonstrated [19,31,44,61–63].

Along with these unanticipated AHLs, we identified production of a rare AHL, 3-oxo-C7-HL, in *P*. *stewartii* and *E*. *carotovora*. We confirmed the structure of this uncommon AHL based on its MS/MS fragmentation spectra (in combination with ^13^C- and ^15^N-isotope labeling). AHLs usually have an even number of carbons in the side-chain as they are constructed by LuxI-type enzymes from fatty acid-derived building blocks (i.e., as acylated acyl carrier proteins), but this AHL has an odd number of carbons. Further studies are required to determine the origins and physiological relevance/function of this type of AHL in these and other bacteria. Only few bacteria have been reported to produce AHLs with side-chains consisting of odd number of carbons [38,40–42,64,65].

### AHL degradation

As the last stage of our study, we investigated degradation of 15 AHLs, both native and non-native, in eight different bacteria using our MS method. Five of these bacteria (i.e., *B*. *cepacia*, *B*. *subtilis*, *E*. *carotovora*, *P*. *aeruginosa*, and *P*. *aureofaciens*) were found capable of degrading AHLs, and each degraded a different set of AHLs at distinct rates. *E*. *coli*, *E*. *herbicola*, and *P*. *stewartii*, in contrast, did not degrade any AHLs. In general, long-chain AHLs were preferentially degraded vs. short-chain AHLs in all degrading microbes, and none were found to degrade non-native AHLs. The latter result bodes well for the use of such non-native AHLs as chemical probes to study QS processes in these bacteria [66]. Of the five bacteria with AHL degradation activity, only *B*. *subtilis* and *P*. *aeruginosa* are known to contain AHL degrading enzymes. *P*. *aeruginosa* was shown to degrade long-chain AHLs (including 3-oxo-C12-HL), but not short-chain AHLs, which conforms to our results [54–56]. *B*. *subtilis* and few other *Bacillus* sp. contain AHL lactonase that has activity against many AHLs, regardless of length of AHL [58,59]. This is in contrast to our results, as we observed no activity against short-chain AHLs in *B*. *subtilis*.

To close, we predict the LC-MS/MS method developed in this study should prove useful for non-targeted identification and quantitation of known AHLs and the discovery of novel AHLs produced by bacteria; the increasing availability of hybrid quadrupole/mass spectrometers should widen and facilitate its implementation.

## Materials and Methods

### Chemicals and AHL standard mix

The AHL standards *N*-heptanoyl-L-homoserine lactone (C7-HL) and *N*-3-(oxododecanoyl)-L-homoserine lactone (3-oxo-C12-HL) were purchased from Sigma-Aldrich. The naturally occurring AHLs *N*-3-(oxotetradecanoyl)-7,8-*cis*-L-homoserine lactone (3-oxo-7,8-*cis*-C14-HL), *N*-3-(oxohexadecanoyl)-11,12-*cis*-L-homoserine lactone (3-oxo-11,12-*cis*-C16-HL), *N*-9,10-*cis*-octadec-L-homoserine lactone (9,10-*cis*-C18-HL), *N*-9,10-*cis*-tetradec-L-homoserine lactone (9,10-*cis*-C14-HL), *N*-3-hydroxydecanoyl-L-homoserine lactone (3-OH-C10-HL), *N*-butyryl-L-homoserine lactone (C4-HL), *N*-octanoyl-L-homoserine lactone (C8-HL), *N*-3-(oxodecanoyl)-L-homoserine lactone (3-oxo-C10-HL), *N*-hexanoyl-L-homoserine lactone (C6-HL), and *N*-dodecanoyl-L-homoserine lactone (C12-HL) were purchased from Cayman. The naturally occurring AHLs *N*-3-(oxohexanoyl)-L-homoserine lactone (3-oxo-C6-HL), *N*-3-(oxooctanoyl)-L-homoserine lactone (3-oxo-C8-HL), and *N*-decanoyl-L-homoserine lactone (C10-HL) and the non-native AHL derivatives C1, C2, C10, C13, R5, S2, S4, mBTL, and ctrl6 were synthesized as previously described using standard solution-phase amide coupling reactions [67,68]. The chemical structures of all AHLs are listed in S1 Table. HPLC-grade acetonitrile, methanol, and ethyl acetate were purchased from Fluka. Dimethyl sulfoxide (DMSO) was purchased from Sigma-Aldrich. HPLC-grade water was purchased from Fisher Scientific. Acetic acid was purchased from Chem-Impex International. Initial stocks were made by dissolving AHL standards into DMSO and further dilutions were done in HPLC-grade water.

### Bacterial strains and growth

*Burkholderia cepacia* AMMO, *Erwinia carotovora*, *Erwinia herbicola* LS00*5*, *Pantoea stewartii*, *Pseudomonas aureofaciens* 30–84 were a gift from Michael Thomas at UW-Madison. *Pseudomonas aeruginosa* PAO1 was provided by Helen Blackwell at UW-Madison. *Edwardsiella tarda* was provided by Federico Rey at UW-Madison. *Bacillus subtilis* 168 was provided by Jade Wang at UW-Madison. *Rhodobacter sphaeroides* was provided by Timothy Donohue at UW-Madison.

Screening of AHL production by *B*. *cepacia*, *E*. *carotovora*, *E*. *herbicola*, *P*. *stewartii*, *P*. *aureofaciens*, *P*. *aeruginosa*, and *B*. *subtilis* was performed in six different carbon sources (1% w/v glucose, 10 mM citrate, 0.5% w/v glycerol, 0.2% w/v acetate, 0.2% w/v mannitol, or 10 mM succinate) using two different salt compositions (AB or M9). AB medium contained 0.4 g/l (NH_4_)_2_SO_4_, 1.2 g/l Na_2_HPO_4_, 3 g/l KH_2_PO_4_, 3 g/l NaCl, 0.1 mM CaCl_2_, 1 mM MgCl_2_, and 3 μM FeCl_3_. M9 medium contained 12.8 g/L Na_2_HPO_4_.7H_2_O, 3 g/l KH_2_PO_4_, 0.5 g/l NaCl, 1 g/l NH_4_Cl, 2 mM MgSO_4_, and 0.1 mM CaCl_2_. Bacterial cultures were started by inoculating a single colony from an LB plate into 5 mL of LB media and grown for 24 hours at 28° C. Next, a 1/1000 dilution of the LB overnight culture was done into minimal media and grown aerobically at 28° C with constant shaking (200 rpm). Spent media samples for AHL quantitation were collected at various time points during growth (i.e., exponential and stationary phase) via centrifugation (14000 rpm, 10 min) and stored at -80° C until further analysis. Samples for AHL quantitation in *E*. *tarda* and *R*. *sphaeroides* were collected similarly with the following exceptions: *E*. *tarda* was grown anaerobically in mega media at 37°C as previously described [69]. *R*. *sphaeroides* was grown anaerobically in SIS media under incandescent light illumination using succinate as carbon source as previously described [70]. All samples for AHL quantitation were collected at stationary phase in triplicate from three parallel cultures.

### Sample preparation

For HPLC-MS analysis, liquid-to-liquid extraction was performed on spent media. After thawing, 250 μL of spent media was extracted three times with 500 μL of ethyl acetate acidified with 0.5% acetic acid. The ethyl acetate phase was collected (total volume 1.5 mL), dried under nitrogen gas, and re-suspended in 250 μL of 20% acetonitrile. Prior to liquid-to-liquid extraction, non-native AHLs S2 and S4 were spiked into samples at concentrations of 3.518 nM and 0.2524 nM, respectively, to correct for extraction efficiency and to serve as internal standards for quantitation. AHLs concentrations were obtained using external calibration curves (S1 Fig) or calculated using internal standards S2 and S4. S3 Table shows the response factors of AHLs against the internal standards S2 and S4 used for quantitation. S3 Fig displays the correlation between AHL quantitation using external calibration curves vs. internal standards.

### HPLC-MS/MS analysis

Aliquots (4 μL) of extracted sample in 20% acetonitrile were subjected to HPLC-MS/MS analysis. HPLC was performed on a Dionex UltiMate 3000 XRS system (Thermo Scientific) using a C18 reverse-phase column (1.7 um particle size, 2.1x50 mm; Acquity UPLC BEH). Solvent A consisted of 10:90 methanol:water with 5 mM ammonium formate and 0.1% formic acid, and solvent B was 100% methanol. The gradient profile for chromatography was as follows: 100% solvent A for 1 min, linear increase in solvent B to 90% over 4 min, isocratic 90% solvent B for 5.5 min, and then equilibration with 100% solvent A for 2 min. The flow rate was constant at 0.2 ml/min.

Compounds separated by HPLC were detected by heated electrospray ionization coupled to high-resolution mass spectroscopy (HESI-MS) (QExactive; Thermo scientific). Analysis was performed under positive ionization mode. Settings for the ion source were: 10 aux gas flow rate, 35 sheath gas flow rate, 1 sweep gas flow rate, 4 μA spray current, 4 kV spray voltage, 320°C capillary temperature, 300°C heater temperature, and 50 S-lens RF level. Nitrogen was used as nebulizing gas by the ion trap source. The MS/MS method was designed to perform an MS1 full-scan (100 to 510 m/z, no fragmentation) together with a series of MS/MS scans (all-ion fragmentation) that divided the m/z range into partially overlapping windows of 40 m/z each. The MS1 full-scan provides data on [M + H]^+^ pseudo-molecular ions, while the MS/MS scans provide corresponding (matched by retention time) fragmentation spectra, all obtained within a single chromatographic run. MS/MS scans (all-ion fragmentation) were centered at 160, 210, 245, 280, 315, 350, 385, 420, 455, 490 m/z using an isolation width of 40.0 m/z. Fragmentations were performed at 17.5, 35, and 52.5 NCE (normalized-collision energy). Mass resolution was set at 35000, AGC target was 1E6, and injection time was 40 ms. Data analysis was performed using the MAVEN software [71] and Thermo Xcalibur software (Thermo scientific).

### AHL hydrolysis of standards

To hydrolyze the lactone ring, AHL standard mix was incubated in 1 M NaOH for 12 hours at room temperature. Hydrolyzed samples were prepared using liquid-to-liquid extraction prior to HPLC-MS analysis as described above.

### Experimental design for AHL degradation

Aliquots (5 mL) of LB broth were inoculated with an isolated colony from an LB plate. After 24 hours, the LB culture was used to start inoculation into 5 mL AB minimal medium with 1% glucose at 1/100 dilution. The AB minimal used in all AHL degradation experiments contained ^15^NH_4_ as the single nitrogen source, this allowed us to differentiate degradation of endogenous AHLs (which are ^15^N-labeled) vs. externally added AHLs. After growing microbes to mid-exponential phase, a variable cocktail mix of AHL standards was added (Fig 5). Media without cells was included as a control in each experiment. Spent media was collected at different time-points, followed by liquid-to-liquid extraction and HPLC-MS/MS analysis as described above. Results from non-inoculated medium and added AHL standard mix were used to correct for spontaneous AHL degradation.

### Genomic search for AHL degrading enzymes

Based on reported lactonases, we collected sequences for the AiiA homologue from *Bacillus* sp. 24B1, Ahld homologue from *Arthrobacter* sp., AhlK homologue from *Klebsiella pneumoniae*, and AttM homologues from *Agrobacterium tumefaciens*. For known acylases, we collected sequences for AiiD from *Ralstonia eutropha*, PvdQ and QuiP from *P*. *aeruginosa*, AhlM from *Streptomyces* sp., and AiiC from *Anabaena* sp. These sequences of known acylases and lactonases were compared against the genomes of *B*. *cepacia*, *B*. *subtilis*, *E*. *carotovora*, *P*. *aeruginosa*, and *P*. *aureofaciens* using BLAST searches.

## Supporting Information

S1 Fig Calibration curves of AHL standards. (PDF)

S2 Fig Fragmentation MS/MS spectra of AHL standards. (PDF)

S3 Fig Correlation between AHL quantitation using internal standard (S2) vs. external calibration curve. Concentration values were calculated for various AHLs in all bacteria using both the quantitation methods. Linear relationship indicates consistency between the two quantitation methods.
(PDF)

S4 Fig Comparison between the mass spectra of 3-oxo-C7-HL against 3-oxo-C6-HL, 3-oxo-C8-HL, and C7-HL. (PDF)

S5 Fig Spectra for no hydrolysis (left panel) and hydrolysis (right panel) of selected AHL standards. Red lines indicate characteristic fragments of the lactone ring of AHLs. Blue line indicates the parent ion. Orange line indicates fragment for the hydrolyzed lactone ring. Structures are provided at the top-right corner of each spectra.
(PDF)

S1 Table Structures, names, and chemical formulas of AHL standards. Standards are indicated as native, non-native, or non-AHL. (PDF)

S2 Table Measured AHLs concentrations in spent media. * *Unanticipated AHLs highlighted in bold. ^†^Concentrations were calculated based on response factors to internal standards S2 and S4. ^‡^Observed mass spectra for unanticipated AHLs matched those of the standards.
(PDF)

S3 Table Response factors of AHL standards vs. internal AHL standards S4 and S2. * *Reported values were calculated as an average of five different concentrations.
(PDF)

## References

[1] Williams P, Winzer K, Chan WC, Cámara M. Look who’s talking: communication and quorum sensing in the bacterial world. Philos Trans R Soc Lond B Biol Sci. 2007;362: 1119–34. 10.1098/rstb.2007.2039 17360280 PMC2435577

[2] Reading NC, Sperandio V. Quorum sensing: the many languages of bacteria. FEMS Microbiol Lett. 2006;254: 1–11. 10.1111/j.1574-6968.2005.00001.x 16451172

[3] Galloway WRJD, Hodgkinson JT, Bowden SD, Welch M, Spring DR. Quorum sensing in Gram-negative bacteria: small-molecule modulation of AHL and AI-2 quorum sensing pathways. Chem Rev. 2011;111: 28–67. 10.1021/cr100109t 21182299

[4] Barnard AML, Bowden SD, Burr T, Coulthurst SJ, Monson RE, Salmond GPC. Quorum sensing, virulence and secondary metabolite production in plant soft-rotting bacteria. Philos Trans R Soc Lond B Biol Sci. 2007;362: 1165–83. 10.1098/rstb.2007.2042 17360277 PMC2435580

[5] Wisniewski-Dyé F, Downie JA. Quorum-sensing in Rhizobium. Antonie Van Leeuwenhoek. 2002;81: 397–407. 12448738 10.1023/a:1020501104051

[6] Venturi V. Regulation of quorum sensing in Pseudomonas. FEMS Microbiol Rev. 2006;30: 274–91. 10.1111/j.1574-6976.2005.00012.x 16472307

[7] Waters CM, Bassler BL. Quorum sensing: cell-to-cell communication in bacteria. Annu Rev Cell Dev Biol. 2005;21: 319–346. 10.1146/annurev.cellbio.21.012704.131001 16212498

[8] Kievit TR De Iglewski BH. Bacterial Quorum Sensing in Pathogenic Relationships. Infect Immun. 2000;68: 4839–4849. 10.1128/IAI.68.9.4839-4849.2000 Updated 10948095 PMC101676

[9] Rutherford ST, Bassler BL, Delany I, Rappuoli R, Seib KL, Ben-tekaya H, et al Bacterial Quorum Sensing: Its Role in Virulence and Possibilities for Its Control. Cold Spring Harb Perspect Med. 2012; 1–26. 10.1101/cshperspect.a012427 23125205 PMC3543102

[10] Nealson KH, Platt T, Hastings JW. Cellular control of the synthesis and activity of the bacterial luminescent system. J Bacteriol. 1970;104: 313–322. 5473898 10.1128/jb.104.1.313-322.1970 PMC248216

[11] Eberhard a, Burlingame a L, Eberhard C, Kenyon GL, Nealson KH, Oppenheimer NJ. Structural identification of autoinducer of Photobacterium fischeri luciferase. Biochemistry. 1981;20: 2444–2449. 10.1021/bi00512a013 7236614

[12] Fuqua WC, Winans SC. A LuxR-LuxI type regulatory system activates Agrobacterium Ti plasmid conjugal transfer in the presence of a plant tumor metabolite. J Bacteriol. 1994;176: 2796–806. Available: http://www.pubmedcentral.nih.gov/articlerender.fcgi?artid=205432&tool=pmcentrez&rendertype=abstract 8188582 10.1128/jb.176.10.2796-2806.1994 PMC205432

[13] Huber B, Riedel K, Hentzer M, Heydorn a, Gotschlich a, Givskov M, et al The cep quorum-sensing system of Burkholderia cepacia H111 controls biofilm formation and swarming motility. Microbiology. 2001;147: 2517–28. Available: http://www.ncbi.nlm.nih.gov/pubmed/11535791 10.1099/00221287-147-9-2517 11535791

[14] Von Bodman SB, Bauer WD, Coplin DL. Quorum sensing in plant-pathogenic bacteria. Annu Rev Phytopathol. 2003;41: 455–82. 10.1146/annurev.phyto.41.052002.095652 12730390

[15] Beck von Bodman S, Farrand SK. Capsular polysaccharide biosynthesis and pathogenicity in Erwinia stewartii require induction by an N-acylhomoserine lactone autoinducer. J Bacteriol. 1995;177: 5000–8. Available: http://www.pubmedcentral.nih.gov/articlerender.fcgi?artid=177277&tool=pmcentrez&rendertype=abstract 7665477 10.1128/jb.177.17.5000-5008.1995 PMC177277

[16] Quiñones B, Dulla G, Lindow SE. Quorum Sensing Regulates Exopolysaccharide Production, Motility, and Virulence in Pseudomonas syringae. Mol Plant-Microbe Interact. 2005;18: 682–693. 10.1094/MPMI-18-0682 16042014

[17] Puskas a, Greenberg EP, Kaplan S, Schaefer a L. A quorum-sensing system in the free-living photosynthetic bacterium Rhodobacter sphaeroides. J Bacteriol. 1997;179: 7530–7. Available: http://www.pubmedcentral.nih.gov/articlerender.fcgi?artid=179706&tool=pmcentrez&rendertype=abstract 9393720 10.1128/jb.179.23.7530-7537.1997 PMC179706

[18] Marketon MM, Glenn SA, Eberhard A, González JE, Gonza JE. Quorum Sensing Controls Exopolysaccharide Production in Sinorhizobium meliloti. J Bacteriol. 2003;185: 325–331. 10.1128/JB.185.1.325 12486070 PMC141839

[19] Steindler L, Venturi V. Detection of quorum-sensing N-acyl homoserine lactone signal molecules by bacterial biosensors. FEMS Microbiol Lett. 2007;266: 1–9. 10.1111/j.1574-6968.2006.00501.x 17233715

[20] Charlesworth J, Kimyon O, Manefield M, Burns BP. Detection and characterization of N-acyl-l-homoserine lactones using GFP-based biosensors in conjunction with thin-layer chromatography. J Microbiol Methods. 2015;118: 164–167. 10.1016/j.mimet.2015.09.012 26407505

[21] Burton EO, Read HW, Pellitteri MC, Hickey WJ. Identification of Acyl-Homoserine Lactone Signal Molecules Produced by Nitrosomonas europaea Strain Schmidt †. 2005;71: 4906–4909. 10.1128/AEM.71.8.4906 16085894 PMC1183371

[22] Wood DW, Gong F, Daykin MM, Williams P, Pierson LS. N-acyl-homoserine lactone-mediated regulation of phenazine gene expression by Pseudomonas aureofaciens 30–84 in the wheat rhizosphere. J Bacteriol. 1997;179: 7663–70. Available: http://www.pubmedcentral.nih.gov/articlerender.fcgi?artid=179727&tool=pmcentrez&rendertype=abstract 9401023 10.1128/jb.179.24.7663-7670.1997 PMC179727

[23] Liu M, Wang H, Griffiths MW. Regulation of alkaline metalloprotease promoter by N-acyl homoserine lactone quorum sensing in Pseudomonas fluorescens. J Appl Microbiol. 2007;103: 2174–84. 10.1111/j.1365-2672.2007.03488.x 18045400

[24] Gould TA, Herman J, Krank J, Murphy RC, Churchill MEA. Specificity of acyl-homoserine lactone synthases examined by mass spectrometry. J Bacteriol. 2006;188: 773–783. 10.1128/JB.188.2.773-783.2006 16385066 PMC1347284

[25] Nieto Penalver CG, Morin D, Cantet F, Saurel O, Milon A, Vorholt J a. Methylobacterium extorquens AM1 produces a novel type of acyl-homoserine lactone with a double unsaturated side chain under methylotrophic growth conditions. FEBS Lett. 2006;580: 561–7. 10.1016/j.febslet.2005.12.062 16412429

[26] Lindemann A, Pessi G, Schaefer AL, Mattmann ME, Christensen QH, Kessler A, et al Isovaleryl-homoserine lactone, an unusual branched-chain quorum-sensing signal from the soybean symbiont Bradyrhizobium japonicum. Proc Natl Acad Sci U S A. 2011;108: 16765–70. 10.1073/pnas.1114125108 21949379 PMC3189028

[27] Purohit a. a., Johansen J a., Hansen H, Leiros HKS, Kashulin a., Karlsen C, et al Presence of acyl-homoserine lactones in 57 members of the Vibrionaceae family. J Appl Microbiol. 2013;115: 835–847. 10.1111/jam.12264 23725044 PMC3910146

[28] Ortori C a, Dubern J-F, Chhabra SR, Cámara M, Hardie K, Williams P, et al Simultaneous quantitative profiling of N-acyl-L-homoserine lactone and 2-alkyl-4(1H)-quinolone families of quorum-sensing signaling molecules using LC-MS/MS. Anal Bioanal Chem. 2011;399: 839–50. 10.1007/s00216-010-4341-0 21046079

[29] Morin D, Grasland B, Vallée-Réhel K, Dufau C, Haras D. On-line high-performance liquid chromatography-mass spectrometric detection and quantification of N-acylhomoserine lactones, quorum sensing signal molecules, in the presence of biological matrices. J Chromatogr A. 2003;1002: 79–92. Available: http://www.ncbi.nlm.nih.gov/pubmed/12885081 10.1016/s0021-9673(03)00730-1 12885081

[30] O’Loughlin CT, Miller LC, Siryaporn A, Drescher K, Semmelhack MF, Bassler BL. A quorum-sensing inhibitor blocks Pseudomonas aeruginosa virulence and biofilm formation. Proc Natl Acad Sci U S A. 2013;110: 17981–17986. 10.1073/pnas.1316981110 24143808 PMC3816427

[31] Han Y, Li X, Qi Z, Zhang X-H, Bossier P. Detection of different quorum-sensing signal molecules in a virulent Edwardsiella tarda strain LTB-4. J Appl Microbiol. 2010;108: 139–47. 10.1111/j.1365-2672.2009.04405.x 19548884

[32] Venturi V, Friscina A, Bertani I, Devescovi G, Aguilar C. Quorum sensing in the Burkholderia cepacia complex. Res Microbiol. 2004;155: 238–244. 10.1016/j.resmic.2004.01.006 15142620

[33] Schu DJ, Carlier AL, Jamison KP, von Bodman S, Stevens AM. Structure/function analysis of the Pantoea stewartii quorum-sensing regulator EsaR as an activator of transcription. J Bacteriol. 2009;191: 7402–9. 10.1128/JB.00994-09 19820098 PMC2786598

[34] Pesci EC, Pearson JP, Seed PC, Iglewski BH. Regulation of las and rhl quorum sensing in Pseudomonas aeruginosa. J Bacteriol. 1997;179: 3127–32. Available: http://www.pubmedcentral.nih.gov/articlerender.fcgi?artid=179088&tool=pmcentrez&rendertype=abstract 9150205 10.1128/jb.179.10.3127-3132.1997 PMC179088

[35] Maddula VSRK, Zhang Z, Pierson E a, Pierson LS. Quorum sensing and phenazines are involved in biofilm formation by Pseudomonas chlororaphis (aureofaciens) strain 30–84. Microb Ecol. 2006;52: 289–301. 10.1007/s00248-006-9064-6 16897305

[36] Zhang Z, Iii LSP. A Second Quorum-Sensing System Regulates Cell Surface Properties but Not Phenazine Antibiotic Production in Pseudomonas aureofaciens. Appl Environ Microbiol. 2001;67: 4305–4315. 10.1128/AEM.67.9.4305 11526037 PMC93161

[37] Kenney DMC, Brown KE, Allison DG. Influence of Pseudomonas aeruginosa exoproducts on virulence factor production in Burkholderia cepacia: evidence of interspecies communication. J Bacteriol. 1995;177: 6989–6992. 7592496 10.1128/jb.177.23.6989-6992.1995 PMC177571

[38] Morohoshi T, Inaba T, Kato N, Kanai K, Ikeda T. Identification of Quorum-Sensing Signal Molecules and the LuxRI Homologs in Fish Pathogen Edwardsiella tarda. J Biosci Bioeng. 2004;98: 274–281. 10.1263/jbb.98.274 16233705

[39] Minogue TD, Wehland-von Trebra M, Bernhard F, von Bodman SB. The autoregulatory role of EsaR, a quorum-sensing regulator in Pantoea stewartii ssp. stewartii: evidence for a repressor function. Mol Microbiol. 2002;44: 1625–35. Available: http://www.ncbi.nlm.nih.gov/pubmed/12067349 10.1046/j.1365-2958.2002.02987.x 12067349

[40] Liu X, Jia J, Popat R, Ortori CA, Li J, Diggle SP, et al Characterisation of two quorum sensing systems in the endophytic Serratia plymuthica strain G3: differential control of motility and biofilm formation according to life-style. BMC Microbiol. 2011;11: 26 10.1186/1471-2180-11-26 21284858 PMC3044098

[41] Liu X, Jia J, Atkinson S, Camara M, Gao K, Li H, et al Biocontrol potential of an endophytic Serratia sp. G3 and its mode of action. World J Microbiol Biotechnol. 2010;26: 1465–1471. 10.1007/s11274-010-0321-y

[42] Ortori CA, Atkinson S, Chhabra SR, Cámara M, Williams P, Barrett DA. Comprehensive profiling of N-acylhomoserine lactones produced by Yersinia pseudotuberculosis using liquid chromatography coupled to hybrid quadrupole-linear ion trap mass spectrometry. Anal Bioanal Chem. 2007;387: 497–511. 10.1007/s00216-006-0710-0 16967185

[43] Dong YH, Wang LH, Xu JL, Zhang HB, Zhang XF, Zhang LH. Quenching quorum-sensing-dependent bacterial infection by an N-acyl homoserine lactonase. Nature. 2001;411: 813–817. 10.1038/35081101 11459062

[44] Yates E a, Philipp B, Buckley C, Atkinson S, Chhabra SR, Sockett RE, et al N-acylhomoserine lactones undergo lactonolysis in a pH-, temperature-, and acyl chain length-dependent manner during growth of Yersinia pseudotuberculosis and Pseudomonas aeruginosa. Infect Immun. 2002;70: 5635–5646. 10.1128/IAI.70.10.5635-5646.2002 12228292 PMC128322

[45] Palmer AG, Senechal AC, Mukherjee A, Ané J-M, Blackwell HE. Plant Responses to Bacterial N-Acyl l-Homoserine Lactones are Dependent on Enzymatic Degradation to l-Homoserine. ACS Chem Biol. 2014;9: 1834–45. 10.1021/cb500191a 24918118 PMC4136694

[46] Medina-Martínez MS, Uyttendaele M, Rajkovic A, Nadal P, Debevere J. Degradation of N-acyl-L-homoserine lactones by Bacillus cereus in culture media and pork extract. Appl Environ Microbiol. 2007;73: 2329–2332. 10.1128/AEM.01993-06 17293532 PMC1855642

[47] Czajkowski R, Jafra S. Quenching of acyl-homoserine lactone-dependent quorum sensing by enzymatic disruption of signal molecules. Acta Biochim Pol. 2009;56: 1–16. 19287806

[48] Uroz S, Heinonsalo J. Degradation of N-acyl homoserine lactone quorum sensing signal molecules by forest root-associated fungi. FEMS Microbiol Ecol. 2008;65: 271–278. 10.1111/j.1574-6941.2008.00477.x 18400006

[49] Wang LH, Weng LX, Dong YH, Zhang LH. Specificity and Enzyme Kinetics of the Quorum-quenching N-Acyl Homoserine Lactone Lactonase (AHL-lactonase). J Biol Chem. 2004;279: 13645–13651. 10.1074/jbc.M311194200 14734559

[50] Dong Y-H, Zhang L-H. Quorum sensing and quorum-quenching enzymes. J Microbiol. 2005;43: 101–109. 2133 [pii] 15765063

[51] Hong KW, Koh CL, Sam CK, Yin WF, Chan KG. Quorum quenching revisited-from signal decays to signalling confusion. Sensors. 2012;12: 4661–4696. 10.3390/s120404661 22666051 PMC3355433

[52] Fetzner S. Quorum quenching enzymes. J Biotechnol. Elsevier B.V.; 2014;201: 2–14. 10.1016/j.jbiotec.2014.09.001 25220028

[53] Grandclement C, Tannieres M, Morera S, Dessaux Y, Faure D. Quorum quenching: Role in nature and applied developments. FEMS Microbiol Rev. 2015;40: 86–116. 10.1093/femsre/fuv038 26432822

[54] Chen F, Gao Y, Chen X, Yu Z, Li X. Quorum quenching enzymes and their application in degrading signal molecules to block quorum sensing-dependent infection. Int J Mol Sci. 2013;14: 17477–17500. 10.3390/ijms140917477 24065091 PMC3794736

[55] Huang JJ, Han J-I, Zhang L-H, Leadbetter JR. Utilization of acyl-homoserine lactone quorum signals for growth by a soil pseudomonad and Pseudomonas aeruginosa PAO1. Appl Environ Microbiol. 2003;69: 5941–5949. 10.1128/AEM.69.10.5941 14532048 PMC201243

[56] Sio CF, Otten LG, Cool RH, Stephen P, Braun PG, Bos R, et al Quorum Quenching by an N -Acyl-Homoserine Lactone Acylase from Pseudomonas aeruginosa PAO1. Infect Immun. 2006;74: 1673–1682. 10.1128/IAI.74.3.1673 16495538 PMC1418629

[57] Wahjudi M, Papaioannou E, Hendrawati O, van Assen AHG, van Merkerk R, Cool RH, et al PA0305 of pseudomonas aeruginosa is a quorum quenching acylhomoserine lactone acylase belonging to the Ntn hydrolase superfamily. Microbiology. 2011;157: 2042–2055. 10.1099/mic.0.043935-0 21372094

[58] Dong YH, Xu JL, Li XZ, Zhang LH. AiiA, an enzyme that inactivates the acylhomoserine lactone quorum-sensing signal and attenuates the virulence of Erwinia carotovora. Proc Natl Acad Sci U S A. 2000;97: 3526–3531. 10.1073/pnas.97.7.3526 10716724 PMC16273

[59] Pan J, Huang T, Yao F, Huang Z, Powell C a., Qiu S, et al Expression and characterization of aiiA gene from Bacillus subtilis BS-1. Microbiol Res. 2008;163: 711–716. 10.1016/j.micres.2007.12.002 18261893

[60] Zhang H-B, Wang L-H, Zhang L-H. Genetic control of quorum-sensing signal turnover in Agrobacterium tumefaciens. Proc Natl Acad Sci U S A. 2002;99: 4638–43. 10.1073/pnas.022056699 11930013 PMC123700

[61] Zhu J, Beaber JW, Moré MI, Fuqua C, Eberhard A, Winans SC. Analogs of the autoinducer 3-oxooctanoyl-homoserine lactone strongly inhibit activity of the TraR protein of Agrobacterium tumefaciens. J Bacteriol. 1998;180: 5398–5405. 9765571 10.1128/jb.180.20.5398-5405.1998 PMC107588

[62] Jones J, Chhabra SR, Downie JA. raiIR Genes Are Part of a Quorum-Sensing Network Controlled by cinI and cinR in Rhizobium leguminosarum. J Bacteriol. 2002;184: 1597–1606. 10.1128/JB.184.6.1597 11872711 PMC134902

[63] Steidle A, Allesen-holm M, Riedel K, Givskov M, Molin S, Eberl L, et al Identification and Characterization of an N Quorum-Sensing System in Pseudomonas putida Strain IsoF. Appl Environ Microbiol. 2002;68: 6371–6382. 10.1128/AEM.68.12.6371 12450862 PMC134430

[64] Lithgow JK, Wilkinson A, Hardman A, Rodelas B, Wisniewski-Dyé F, Williams P, et al The regulatory locus cinRI in Rhizobium leguminosarum controls a network of quorum-sensing loci. Mol Microbiol. 2000;37: 81–97. 10.1046/j.1365-2958.2000.01960.x 10931307

[65] Horng YT, Deng SC, Daykin M, Soo PC, Wei JR, Luh KT, et al The LuxR family protein SpnR functions as a negative regulator of N-acylhomoserine lactone-dependent quorum sensing in Serratia marcescens. Mol Microbiol. 2002;45: 1655–1671. 10.1046/j.1365-2958.2002.03117.x 12354232

[66] Welsh MA, Blackwell HE. Chemical probes of quorum sensing: from compound development to biological discovery. FEMS Microbiol Rev. 2016; 10.1093/femsre/fuw009 27268906 PMC5007280

[67] Moore JD, Rossi FM, Welsh MA, Nyffeler KE, Blackwell HE. A Comparative Analysis of Synthetic Quorum Sensing Modulators in Pseudomonas aeruginosa: New Insights into Mechanism, Active Efflux Susceptibility, Phenotypic Response, and Next-Generation Ligand Design. J Am Chem Soc. 2015;137: 14626–39. 10.1021/jacs.5b06728 26491787 PMC4665086

[68] Geske GD, O’Neill JC, Miller DM, Mattmann ME, Blackwell HE. Modulation of bacterial quorum sensing with synthetic ligands: systematic evaluation of N-acylated homoserine lactones in multiple species and new insights into their mechanisms of action. J Am Chem Soc. 2007;129: 13613–25. 10.1021/ja074135h 17927181 PMC2592086

[69] Romano KA, Vivas EI, Amador-noguez D, Rey FE. Intestinal Microbiota Composition Modulates Choline Bioavailability from Diet and Accumulation of the Proatherogenic Metabolite Trimethylamine-N-Oxide. MBio. 2015;6: 1–14. 10.1128/mBio.02481-14 Editor 25784704 PMC4453578

[70] Sistrom WR. A Requirement for Sodium in the Growth of Rhodopseudomonas spheroides. J Gen Microbiol. 1960;22: 778–785. 10.1099/00221287-22-3-778 14447230

[71] Clasquin MF, Melamud E, Rabinowitz JD. LC-MS data processing with MAVEN: a metabolomic analysis and visualization engine. Curr Protoc Bioinformatics. 2012;Chapter 14: Unit14.11. 10.1002/0471250953.bi1411s37 22389014 PMC4055029

