# Supplementary figures and images for: Identification of Unanticipated and Novel N-Acyl L-Homoserine Lactones (AHLs) Using a Sensitive Non-Targeted LC-MS/MS Method
Source: PLoS One. 2016 Oct 5;11(10):e0163469. doi: 10.1371/journal.pone.0163469 (PMC5051804; doi:10.1371/journal.pone.0163469)

Intensity

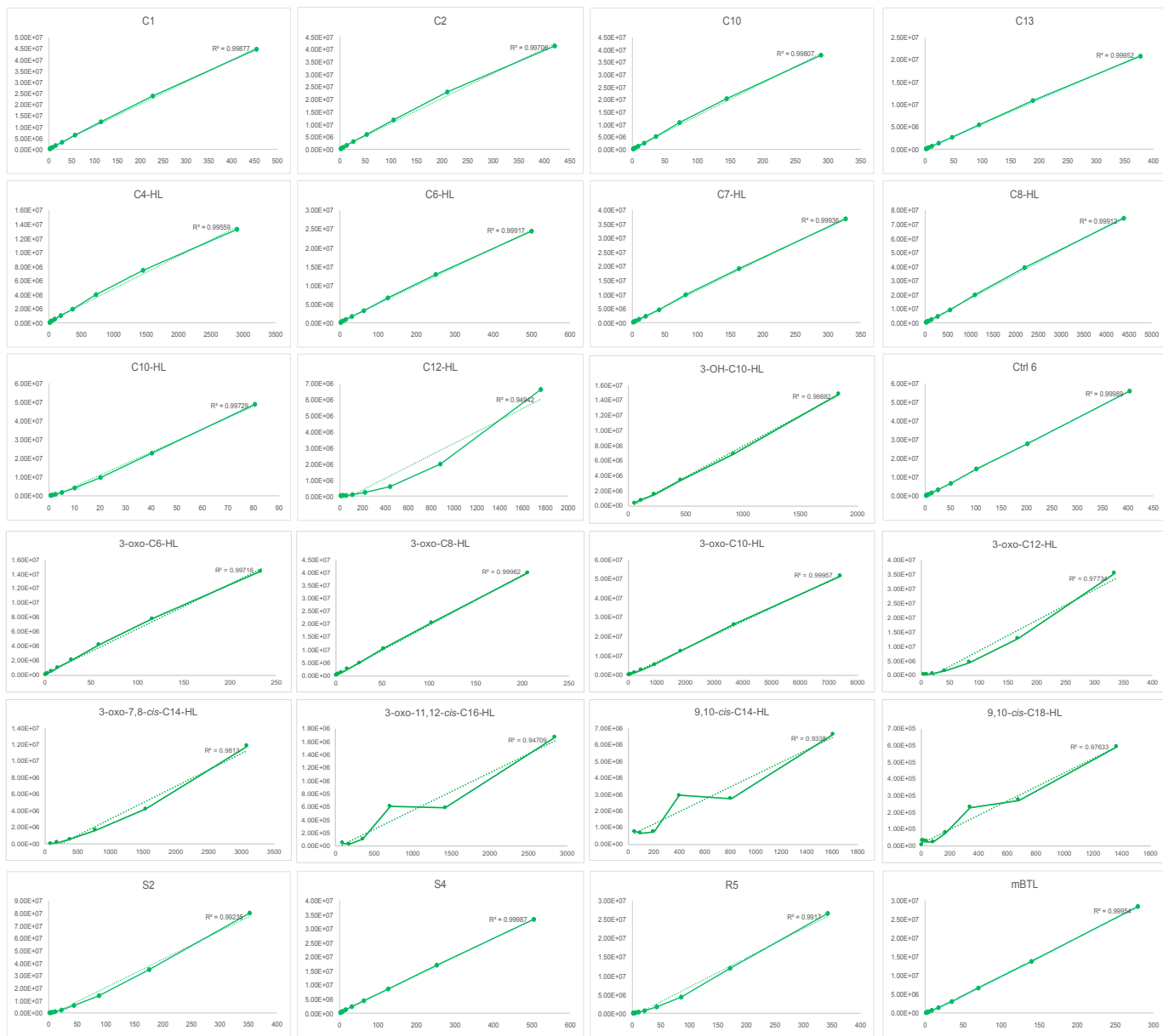

Concentration (nM)

**S1 Fig: Calibration curves of AHL standards.**

Supplement: S1 Fig — (PDF) [file pone.0163469.s001.pdf]
