# Supplementary material for: Identification of Unanticipated and Novel N-Acyl L-Homoserine Lactones (AHLs) Using a Sensitive Non-Targeted LC-MS/MS Method
Source: PLoS One. 2016 Oct 5;11(10):e0163469. doi: 10.1371/journal.pone.0163469 (PMC5051804; doi:10.1371/journal.pone.0163469)
Supplement: S2 Fig — (PDF) [file pone.0163469.s002.pdf]

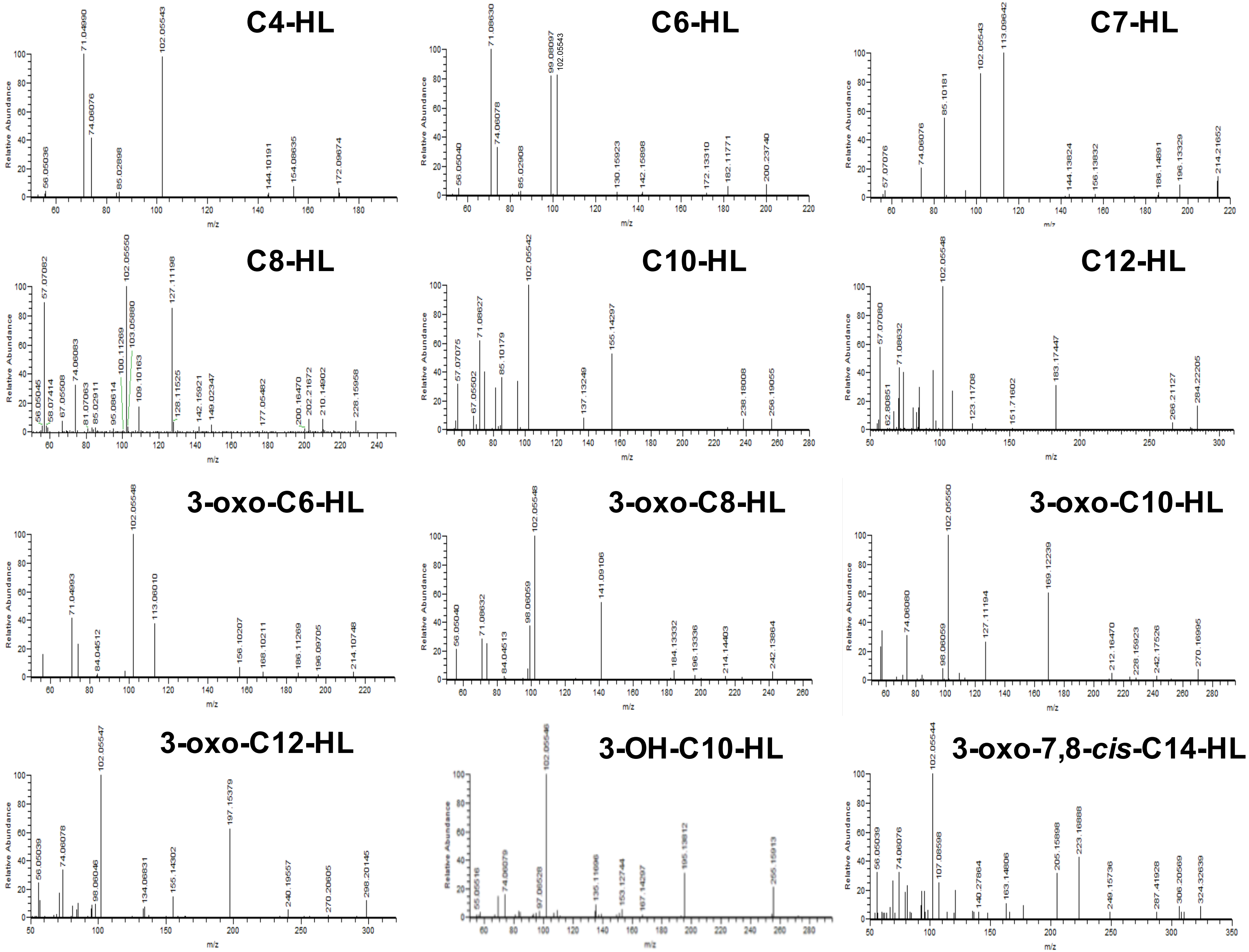

S2 Fig: Fragmentation MS/MS spectra of AHL standards (1).

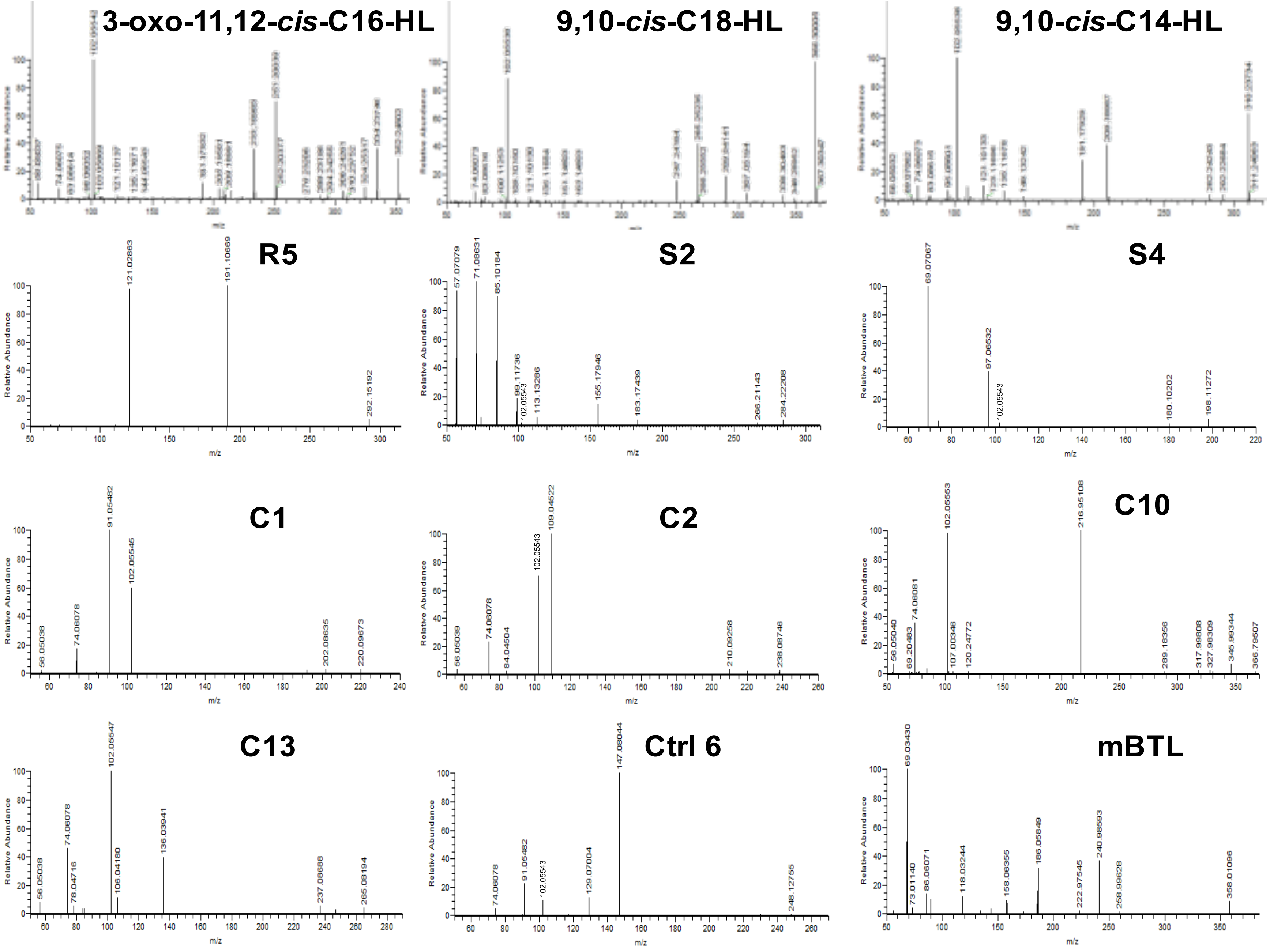

S2 Fig: Fragmentation MS/MS spectra of AHL standards (2).
