# Supplementary material for: Identification of Unanticipated and Novel N-Acyl L-Homoserine Lactones (AHLs) Using a Sensitive Non-Targeted LC-MS/MS Method
Source: PLoS One. 2016 Oct 5;11(10):e0163469. doi: 10.1371/journal.pone.0163469 (PMC5051804; doi:10.1371/journal.pone.0163469)
Supplement: S3 Fig — Concentration values were calculated for various AHLs in all bacteria using both the quantitation methods. Linear relationship indicates consistency between the two quantitation methods. (PDF) [file pone.0163469.s003.pdf]

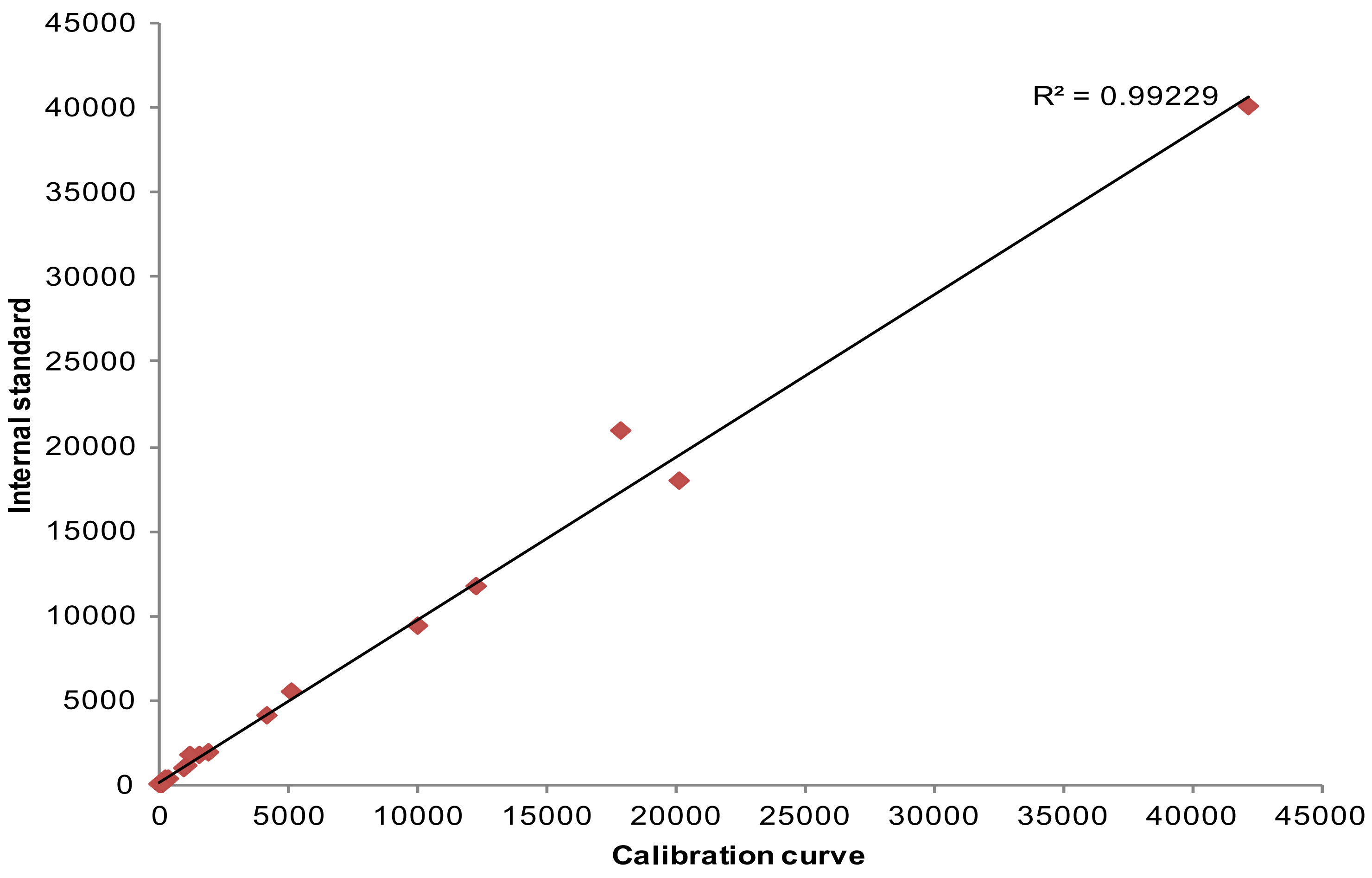

S3 Fig: Correlation between AHL quantitation using internal standard (S2) vs. external calibration curve. Concentration values were calculated for various AHLs in all bacteria using both the quantitation methods. Linear relationship indicates consistency between the two quantitation methods.
