# Supplementary material for: Identification of Unanticipated and Novel N-Acyl L-Homoserine Lactones (AHLs) Using a Sensitive Non-Targeted LC-MS/MS Method
Source: PLoS One. 2016 Oct 5;11(10):e0163469. doi: 10.1371/journal.pone.0163469 (PMC5051804; doi:10.1371/journal.pone.0163469)
Supplement: S5 Fig — Red lines indicate characteristic fragments of the lactone ring of AHLs. Blue line indicates the parent ion. Orange line indicates fragment for the hydrolyzed lactone ring. Structures are provided at the top-right corner of each spectra. (PDF) [file pone.0163469.s005.pdf]

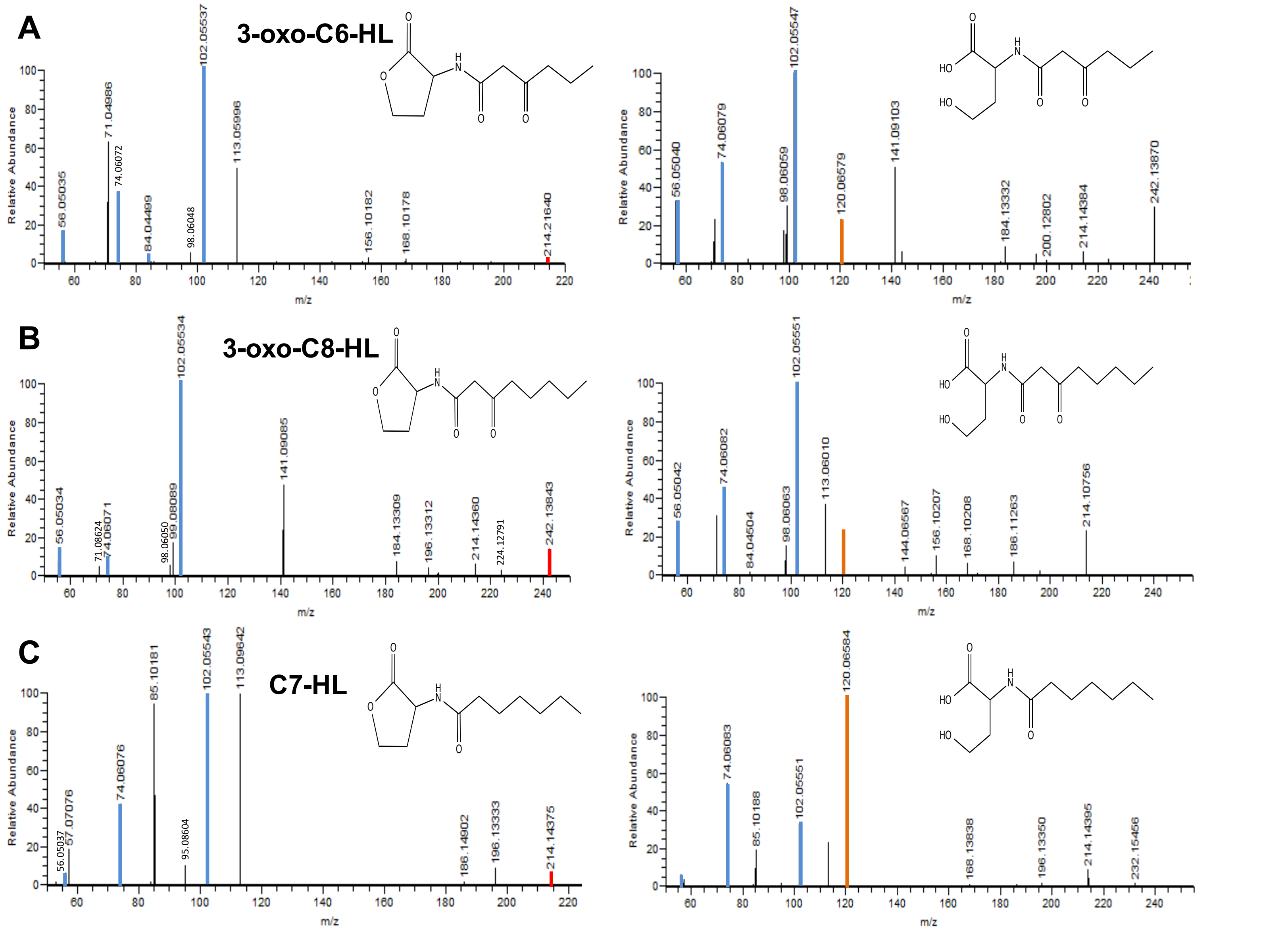

S5 Fig: Spectra for no hydrolysis (left panel) and hydrolysis (right panel) of selected AHL standards. Red lines indicate characteristic fragments of the lactone ring of AHLs. Blue line indicates the parent ion. Orange line indicates fragment for the hydrolyzed lactone ring. Structures are provided at the top-right corner of each spectra.
