# Supplementary material for: Identification of Unanticipated and Novel N-Acyl L-Homoserine Lactones (AHLs) Using a Sensitive Non-Targeted LC-MS/MS Method
Source: PLoS One. 2016 Oct 5;11(10):e0163469. doi: 10.1371/journal.pone.0163469 (PMC5051804; doi:10.1371/journal.pone.0163469)
Supplement: S1 Table — (PDF) [file pone.0163469.s006.pdf]

S1 Table: Structures, names, and chemical formulas of AHL standards. Standards are indicated as native, non-native, or non-AHL.

Name<br>Property	Structure	Chemical formula
C4-HL<br>Native	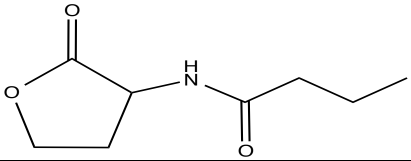	$C_8H_{13}NO_3$
C6-HL<br>Native	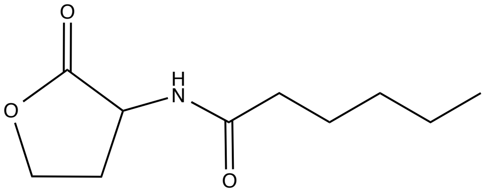	$C_{10}H_{17}NO_3$
3-oxo-C6-HL<br>Native	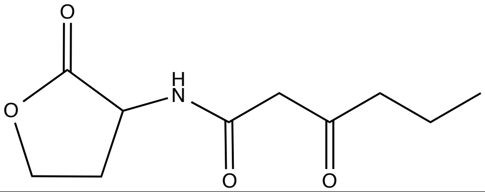	$C_{10}H_{15}NO_4$
C7-HL<br>Native	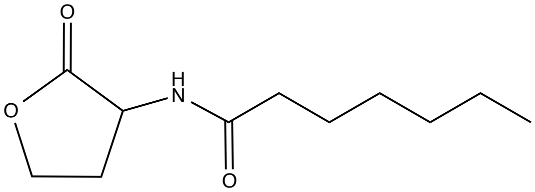	$C_{11}H_{19}NO_3$
C8-HL<br>Native	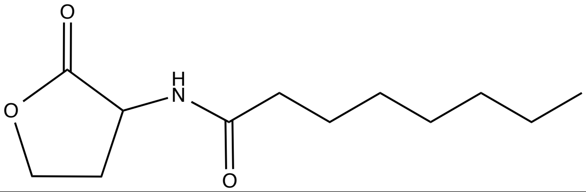	$C_{12}H_{21}NO_3$
3-oxo-C8-HL<br>Native	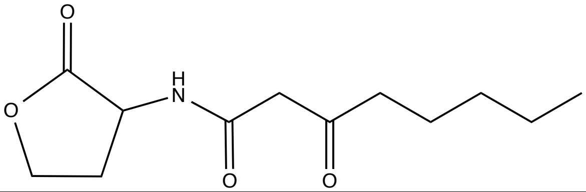	$C_{12}H_{19}NO_4$
C10-HL<br>Native	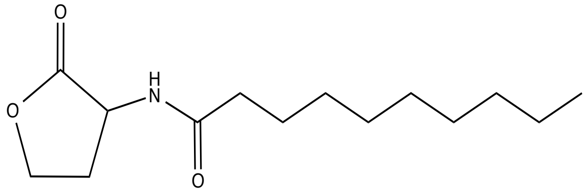	$C_{14}H_{25}NO_3$
3-oxo-C10-HL<br>Native	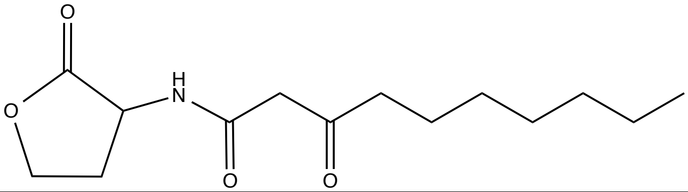	$C_{14}H_{23}NO_4$

3-OH-C10-HL<br>Native	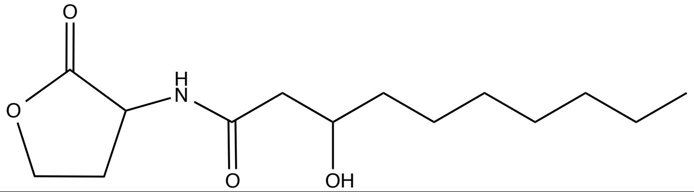	$C_{14}H_{25}NO_4$
C12-HL<br>Native	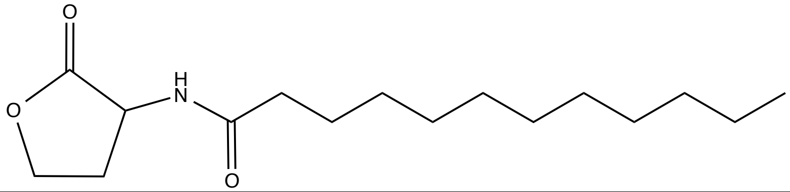	$C_{16}H_{29}NO_3$
3-oxo-C12-HL<br>Native	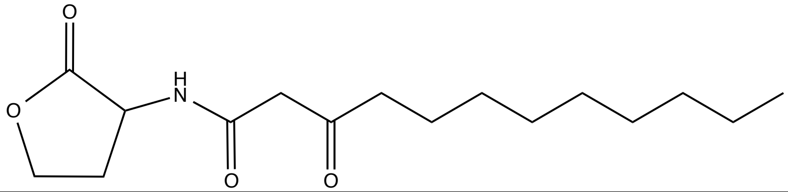	$C_{16}H_{27}NO_4$
9,10-*cis*-C14-HL<br>Native	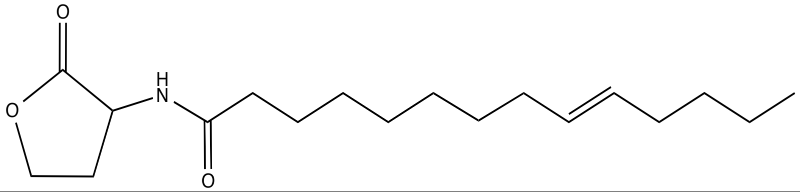	$C_{18}H_{31}NO_3$
3-oxo-7,8-*cis*-C14-HL<br>Native	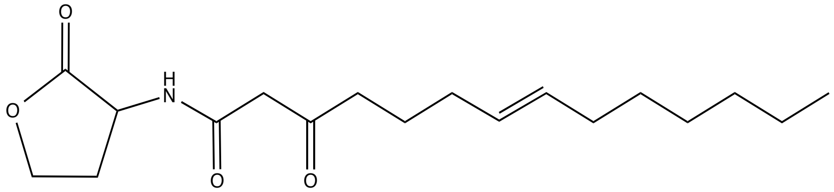	$C_{18}H_{29}NO_4$
3-oxo-11,12-*cis*-C16-HL<br>Native	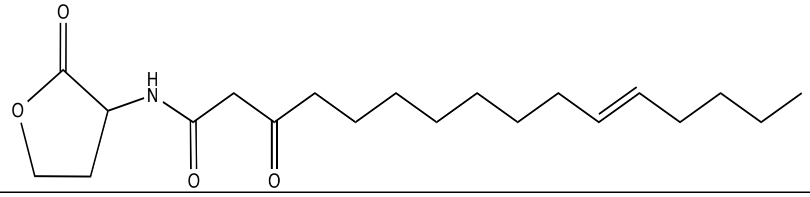	$C_{20}H_{33}NO_4$
9,10-*cis*-C18-HL<br>Native	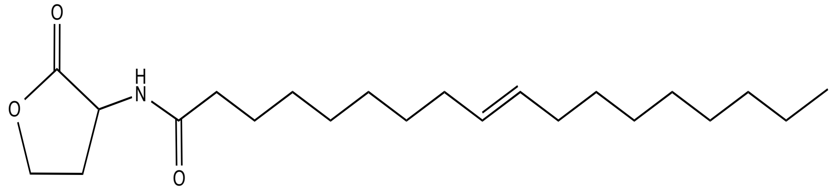	$C_{22}H_{39}NO_3$
C1<br>Non-native	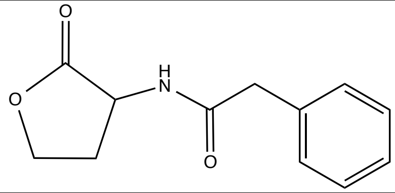	$C_{12}H_{13}NO_3$

C2<br>Non-native	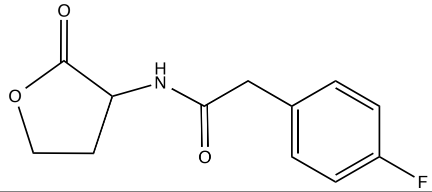	$C_{12}H_{12}FNO_3$
C10<br>Non-native	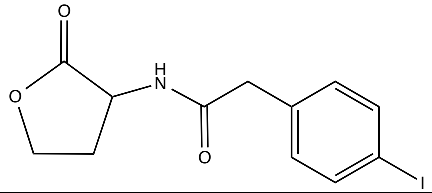	$C_{12}H_{12}INO_3$
C13<br>Non-native	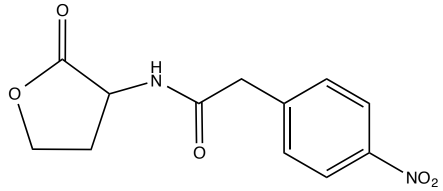	$C_{12}H_{12}IN_2O_5$
S2<br>Non-native	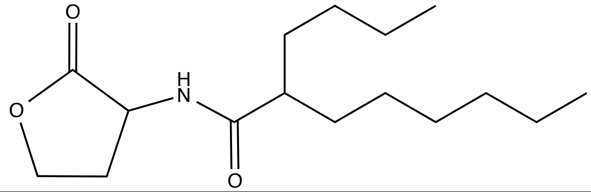	$C_{16}H_{29}NO_3$
S4<br>Non-native	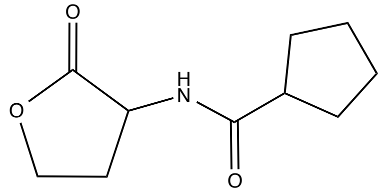	$C_{10}H_{15}NO_3$
R5<br>Non-native	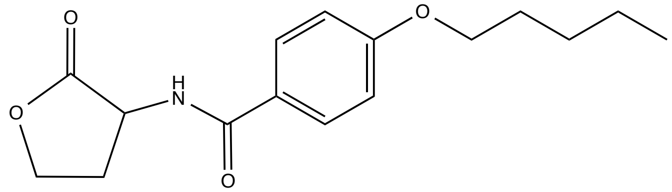	$C_{16}H_{21}NO_4$
Ctrl6<br>Non-native	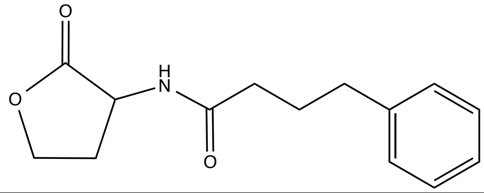	$C_{14}H_{17}NO_3$
mBTL<br>Non-AHL	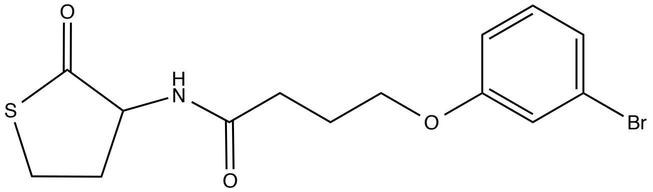	$C_{14}H_{16}BrNO_3S$
