# Supplementary material for: Identification of Unanticipated and Novel N-Acyl L-Homoserine Lactones (AHLs) Using a Sensitive Non-Targeted LC-MS/MS Method
Source: PLoS One. 2016 Oct 5;11(10):e0163469. doi: 10.1371/journal.pone.0163469 (PMC5051804; doi:10.1371/journal.pone.0163469)
Supplement: S2 Table — * *Unanticipated AHLs highlighted in bold. †Concentrations were calculated based on response factors to internal standards S2 and S4. ‡Observed mass spectra for unanticipated AHLs matched those of the standards. (PDF) [file pone.0163469.s007.pdf]

S2 Table: Measured AHLs concentrations in spent media.*,†,‡

			Concentration (nM)		Mass (m/z)		Retention time (min)	
**Bacterium**	**Media**	**AHL**	**S2**	**S4**	**Expected**	**Observed**	**Expected**	**Observed**
B. cepacia	**AB w/ 1% glucose**	**3-oxo-C10-HL**	34.5 ± 4.8	36.4 ± 2.0	270.170	270.169	6.14	6.15
		**3-oxo-C8-HL**	1.3 ± 0.2	1.4 ± 0.1	242.138	242.138	5.22	5.23
		**3-OH-C10-HL**	1745.9 ± 310.5	1893.4 ± 129.1	272.185	272.186	6.21	6.21
		**C4-HL**	72.1 ± 11.8	73.2 ± 2.7	172.097	172.096	1.91	1.92
		C8-HL	25387.2 ± 3502.9	26096.1 ± 525.9				
		C6-HL	147.52 ± 24.5	150.9 ± 6.2				
		**C7-HL**	3.8 ± 0.4	3.9 ± 0.1	214.144	214.143	5.58	5.58
		**C10-HL**	2.2	2.6	256.190	256.190	6.70	6.40
		**3-oxo-C12-HL**	3.1 ± 2.6	3.5 ± 2.8	298.201	298.201	6.78	6.68
E. tarda	**Mega-media**	3-oxo-C6-HL	115.2 ± 5.9	128.5 ± 3.7				
		**C8-HL**	42.5 ± 0.8	47.9 ± 1.3	228.159	228.159	6.03	6.03
		C6-HL	57.3 ± 5.2	64.2 ± 4.1				
		C4-HL	12.8 ± 0.5	14.2 ± 0.1				
		**3-oxo-C8-HL**	3.7 ± 0.03	4.3 ± 0.1	242.138	242.138	5.22	5.23
E. carotovora	**AB w/ 1% glucose**	**3-oxo-C10-HL**	1229.7 ± 53.2	1216.6 ± 69.6	270.170	270.170	6.14	6.14
		**3-oxo-C8-HL**	3777.9 ± 102.7	3686.0 ± 186.3	242.138	242.139	5.22	5.23
		3-oxo-C6-HL	165.3 ± 3.1	158.2 ± 10.5				
		**C7-HL**	42.0 ± 1.3	40.8 ± 2.6	214.144	214.144	5.58	5.58
		**C8-HL**	9167.9 ± 543.6	8837.5 ± 661.0	228.159	228.159	6.03	6.03
		**C6-HL**	29.9 ± 0.8	28.8 ± 2.4	200.128	200.128	4.95	4.96
E. herbicola	**AB w/ 1% glucose**	C4-HL	6267.6 ± 302.4	6010.0 ± 68.4				
		**C8-HL**	5.2 ± 1.0	5.0 ± 1.2	228.159	228.158	6.03	6.01
		**3-oxo-C10-HL**	7.6 ± 1.3	7.5 ± 0.9	270.170	270.169	6.14	6.08
P. stewartii	**AB w/ 0.5% glycerol**	**3-oxo-C10-HL**	49.5 ± 1.8	49.0 ± 1.1	270.170	270.169	6.14	6.14
		**3-oxo-C8-HL**	217.9 ± 5.8	212.8 ± 9.0	242.138	242.138	5.22	5.23
		3-oxo-C6-HL	11912.3 ± 517.3	11395.3 ± 100.8				
		**C7-HL**	1.2 ± 0.3	1.1 ± 0.2	214.144	214.143	5.58	5.59
		**C8-HL**	94.2 ± 5.1	90.8 ± 2.5	228.159	228.159	6.03	6.03
		**C6-HL**	666.0 ± 36.5	640.1 ± 9.3	200.128	200.128	4.95	4.96

		**C4-HL**	239.8 ± 14.2	228.7 ± 3.3	172.097	172.096	1.91	1.91
P. aureofaciens	**AB w/ 0.5% glycerol**	C6-HL	8.9 ± 0.4	9.1 ± 0.5				
		**C4-HL**	5130.8 ± 264.6	5171.0 ± 257.5	172.097	172.097	1.91	1.91
		**3-oxo-C10-HL**	5.7 ± 2.7	5.9 ± 2.8	270.170	270.170	6.14	6.10
P. aeruginosa	**AB w/ 1% glucose**	**3-oxo-C10-HL**	10681.8 ± 2179.9	11890.9 ± 700.5	270.170	270.170	6.14	6.13
		**3-oxo-C8-HL**	21.2 ± 4.1	23.2 ± 0.8	242.138	242.138	5.22	5.21
		3-oxo-C6-HL	23.2 ± 4.9	25.0 ± 1.8				
		3-oxo-C12-HL	2334.3 ± 461.7	2958.0 ± 155.0				
		C8-HL	177.3 ± 33.2	192.6 ± 9.8				
		C6-HL	50.4 ± 9.8	54.5 ± 2.6				
		C4-HL	49018.3 ± 10206.2	52630.6 ± 4236.2				
		3-OH-C10-HL	279.7	296.0				
R. sphaeroides	**SIS**	9,10-*cis*-C14-HL	28171.1 ± 7086.5	32303.1 ± 7075.1				

*Unanticipated AHLs highlighted in bold.

†Concentrations were calculated based on response factors to internal standards S2 and S4.

‡Observed mass spectra for unanticipated AHLs matched those of the standards.
