# Supplementary material for: Identification of Unanticipated and Novel N-Acyl L-Homoserine Lactones (AHLs) Using a Sensitive Non-Targeted LC-MS/MS Method
Source: PLoS One. 2016 Oct 5;11(10):e0163469. doi: 10.1371/journal.pone.0163469 (PMC5051804; doi:10.1371/journal.pone.0163469)
Supplement: S3 Table — * *Reported values were calculated as an average of five different concentrations. (PDF) [file pone.0163469.s008.pdf]

S3 Table: Response factors of AHL standards vs. internal AHL standards S4 and S2.*

Standard	S4	S2
C4-HL	6.03	16.14
C6-HL	1.37	3.63
3-oxo-C6-HL	1.20	3.21
C7-HL	0.78	2.07
C8-HL	4.08	10.80
3-oxo-C8-HL	0.38	1.00
C10-HL	0.14	0.34
3-OH-C10-HL	7.33	18.37
3-oxo-C10-HL	5.87	15.14
C12-HL	47.94	102.62
3-oxo-C12-HL	0.55	1.25
9,10-*cis*-C14-HL	0.10	27.70
3-oxo-7,8-*cis*-C14-HL	40.12	83.89
3-oxo-11,12-*cis*-C16-HL	164.55	371.23
9,10-*cis*-C18-HL	0.12	2486.77
C1	0.69	1.85
C2	0.77	2.03
C10	0.56	1.48
C13	1.37	3.62
R5	0.98	2.38
Ctrl 6	0.83	2.14
mBTL	0.89	2.17

*Reported values were calculated as an average of five different concentrations.
